# Systemic LINE‐1 RNA in Plasma Extracellular Vesicles Drives Neuroinflammation and Cognitive Dysfunction via cGAS‐STING Pathway in Aging

**DOI:** 10.1111/acel.70350

**Published:** 2026-01-02

**Authors:** Shuyi Yu, Qian Cheng, Qian Yu, Zhikang Cui, Hang Chen, Shuang Wu, Yan Jin, Yunshan Wang, Ming Li, Zhiming Lu

**Affiliations:** ^1^ Department of Clinical Laboratory Shandong Provincial Hospital Affiliated to Shandong First Medical University Jinan China; ^2^ Shandong University Jinan China

**Keywords:** aging, cGAS, extracellular vesicles, LINE‐1, microglia, STING

## Abstract

Aging is characterized by systemic inflammation and progressive cognitive decline, yet the molecular pathways linking peripheral aging signals to central nervous system dysfunction remain elusive. Here, we identify plasma extracellular vesicle (EV)‐derived long interspersed nuclear element‐1 (LINE‐1) RNA as a potent systemic aging factor mediating neuroinflammation and cognitive impairment in humans and mice. Plasma EV LINE‐1 RNA levels markedly increase with age and strongly correlate with established brain aging biomarkers, including neurofilament light chain (NFL). Utilizing mouse models, we demonstrate that EVs from aged individuals penetrate the blood–brain barrier, deliver LINE‐1 RNA to microglia, and initiate cGAS‐STING signaling, leading to pronounced neuroinflammation, neuronal damage, and impaired cognition. Pharmacological blockade of LINE‐1 reverse transcription by 3TC or inhibition of STING signaling with H151 significantly ameliorates these age‐associated deficits. Notably, aged peripheral tissues, especially brain and lung, emerge as primary sources of pro‐aging EVs enriched with LINE‐1 RNA, revealing a novel mechanism of inter‐organ communication in aging. Our findings position EV‐derived LINE‐1 RNA and its downstream cGAS‐STING pathway as critical systemic drivers of brain aging, presenting promising therapeutic targets for mitigating cognitive decline and age‐related neurodegenerative diseases.

## Graphical abstract

Plasma extracellular vesicle‐derived LINE‐1 RNA increases with age and crosses the blood–brain barrier to activate microglial cGAS‐STING signaling, driving neuroinflammation and cognitive decline. Inhibition of LINE‐1 reverse transcription or STING signaling ameliorates these aging‐related deficits. This identifies a key systemic mechanism of brain aging and a potential therapeutic target.

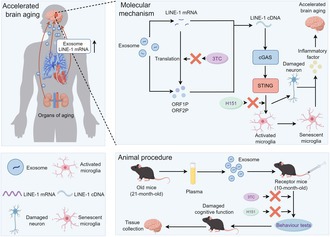

## 1 Introduction

Aging is a systemic process characterized by chronic inflammation, cellular senescence, and functional decline across multiple organs, including the brain (Teissier et al. 2022; Zhang et al. 2022; Lunney 2003). One of the most prominent features of brain aging is neuroinflammation (Stephenson et al. 2018), which is driven by microglial activation and associated with both neuronal damage and cognitive decline (Grabert et al. 2016; Streit et al. 2004). These processes contribute to age‐related brain disorders, such as Alzheimer's disease (AD) and Parkinson's disease (PD) (Gaikwad et al. 2024; Gelders et al. 2018). While it is well established that brain aging is influenced by intrinsic cellular mechanisms (López‐Otín et al. 2023; Allen et al. 2023), the role of systemic aging signals in driving brain dysfunction remains poorly understood.

Extracellular vesicles (EVs), including exosomes, are nano‐sized vesicles secreted by almost all cell types and act as critical mediators of intercellular communication (Doyle and Wang 2019; Kalluri and LeBleu 2020). EVs carry bioactive molecules, including RNA, DNA, proteins, and lipids, and can cross the blood–brain barrier (BBB) to deliver their cargo into the brain (Ramos‐Zaldívar et al. 2022; Ransom et al. 2024). Increasing evidence suggests that EVs play a role in aging and age‐associated diseases by transporting inflammatory signals and other pro‐aging molecules between tissues (Guo et al. 2020; Morales‐Prieto et al. 2022; Li et al. 2018). However, there is currently a lack of systematic understanding of the organ‐specific origin of EVs during aging and their secretory dynamics, particularly the relative contributions of EVs from different tissue sources in systemic aging. Additionally, the specific contributions of EVs to brain aging and the molecular cargo responsible for neuroinflammation remain unclear, limiting our comprehensive understanding of the mechanisms underlying EVs‐mediated inter‐organ communication in the aging process.

The long interspersed nuclear element‐1 (LINE‐1) is an endogenous retrotransposon that comprises approximately 17% of the human genome (Lander et al. 2001). Normally suppressed by epigenetic mechanisms, LINE‐1 activity is increased with age and has been implicated in genomic instability, cellular senescence, and chronic inflammation (Cordaux and Batzer 2009; Della Valle et al. 2022; Miller et al. 2021). Although the age‐dependent activation of LINE‐1 and its role in genomic instability and cellular senescence have been extensively studied, its role in transmitting pro‐aging signals between organs (e.g., peripheral organs and the brain) via EVs remains unknown. LINE‐1 RNA can be reverse‐transcribed into cDNA by its encoded reverse transcriptase (De Cecco et al. 2019; Thawani et al. 2024), which may activate innate immune pathways, including the cyclic GMP‐AMP synthase (cGAS)‐stimulator of interferon genes (STING) signaling pathway (Gamdzyk et al. 2020; Mathavarajah and Dellaire 2024). This pathway, known for its role in detecting cytosolic DNA, triggers inflammation and contributes to cellular senescence (Gulen et al. 2023; Xie et al. 2023; Loo et al. 2020). While LINE‐1 activity is primarily investigated in the context of genomic instability, its systemic effects and potential role in mediating the effects of peripheral organs on brain aging when transported via EVs remain unexplored (Kawamura et al. 2019).

Microglia, the resident immune cells of the brain, are central to neuroinflammation during aging (Wendimu and Hooks 2022; Eyo and Wu 2019). In their normal state, microglia play an essential role in maintaining brain homeostasis by clearing debris and supporting neuronal health (Colonna and Butovsky 2017; Choi et al. 2020). However, during the aging process, microglia exhibit chronic activation and reduced phagocytic capacity, contributing to persistent inflammation and neuronal damage (Leng and Edison 2021; Safaiyan et al. 2021). To date, whether systemic signals, such as EV‐carried LINE‐1 RNA, can trigger microglial activation and promote neuroinflammation has not been fully investigated.

In this study, we aimed to elucidate the role of plasma EV‐derived LINE‐1 RNA as a systemic aging signal and its impact on brain aging. We are the first to systematically investigate the role of LINE‐1 carried by EVs in inter‐organ communication, particularly its potential contribution to brain aging, providing a new perspective on understanding the systemic regulatory mechanisms of aging. We used a combination of molecular, cellular, and in vivo approaches to address the following objectives: (1) to quantify the levels of LINE‐1 RNA in plasma EVs across different age groups in humans to investigate its age‐related enrichment; (2) to evaluate whether old EVs containing LINE‐1 RNA can cross the BBB and activate microglia; (3) to investigate the activation of the cGAS‐STING signaling pathway in microglia upon exposure to EV‐derived LINE‐1 RNA; and (4) to use pharmacological interventions targeting LINE‐1 reverse transcription (via 3TC) and STING signaling (via H151) to assess their effects on neuroinflammation and cognitive function in old EV‐treated mice.

By integrating these approaches, this study seeks to address critical gaps in understanding the systemic origins of brain aging and to identify potential therapeutic strategies targeting EV‐carried LINE‐1 RNA and its downstream inflammatory pathways.

## 2 Results

### 2.1 Age‐Dependent Increase in Plasma EV LINE‐1 RNA and Its Correlation With Brain Aging Biomarkers

**FIGURE 1:**
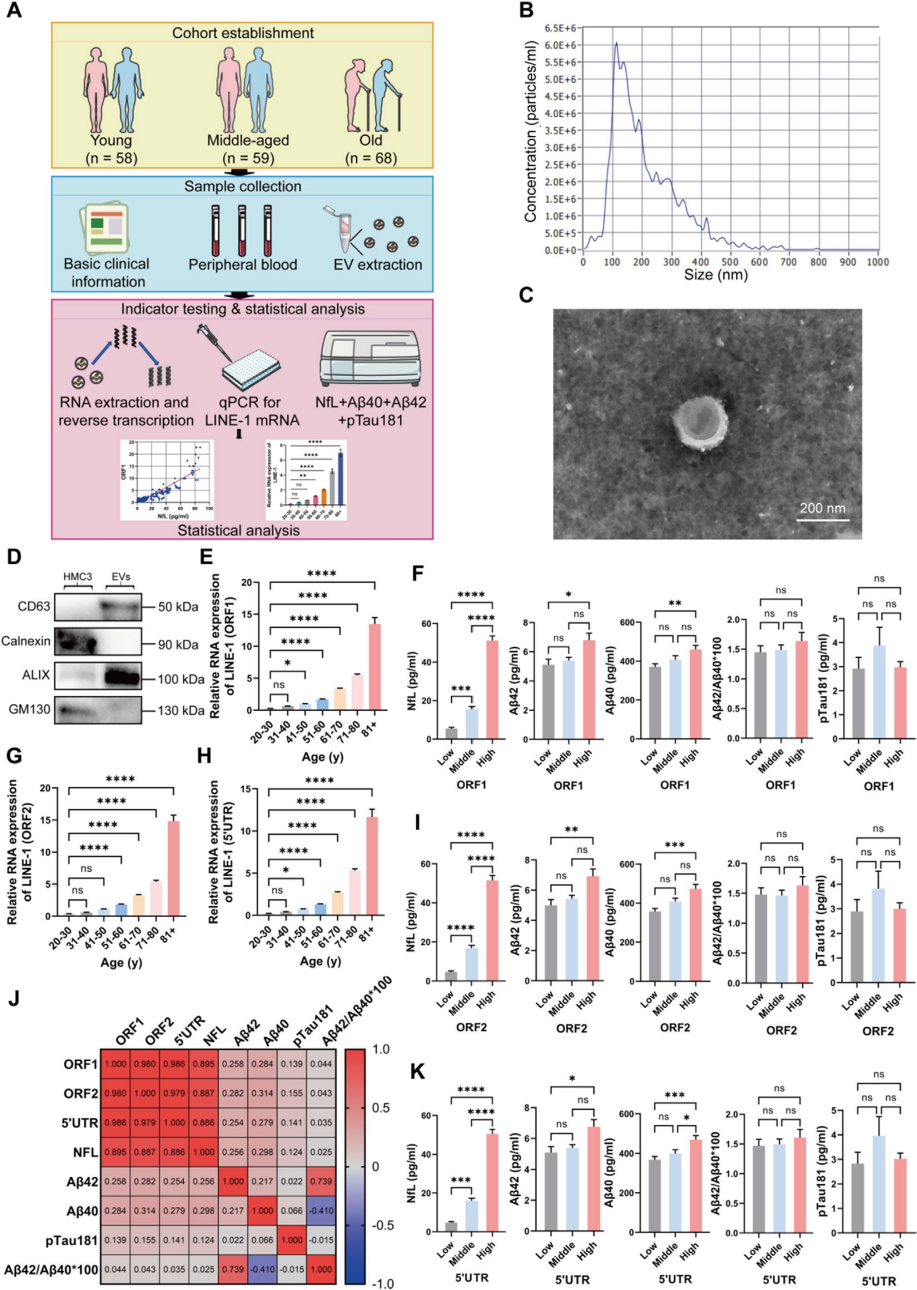
Increased expression of LINE‐1 mRNA in plasma extracellular vesicles (EVs) with age and significantly correlated with brain aging biomarkers. (A) Flow chart of experimental design. (B) Nanoparticle‐tracking analysis (NTA) of plasma EVs. (C) Expected size of EVs and morphology by transmission electron microscopy. (D) Western blotting analysis based on the lysate fractions of HMC3 as control and EVs extracted and measured in (B) and (C) using antibodies against the indicated EV markers with GM130 and Calnexin as negative control markers. (E, G, H) Increased levels of plasma EV LINE‐1 mRNA with age. EVs are isolated from the plasma of 185 individuals presented in Table 1. RNA is extracted and then amplified using qPCR based on three sets of LINE‐1 specific primers. (F, I, K) Expression of brain aging biomarkers in different plasma EV LINE‐1 mRNA tertiles based on three sets of LINE‐1 specific primers (ORF1: Low, < 1.008963092; Middle, 1.008963092–3.765583409; High, > 3.765583409. ORF2: Low, < 1.238225366; Middle, 1.238225366–3.502230212; High, > 3.502230212. 5′UTR: Low, < 0.861948731; Middle, 0.861948731–3.001479813; High, > 3.001479813). One‐way ANOVA is used to analyze the differences between groups for normally distributed data, and non‐normally distributed data are analyzed by non‐parametric tests. Significant difference from control is determined based on *p < 0.05, **p < 0.01, ***p < 0.001, and ****p < 0.0001, respectively; ns, not significant. (J) Spearman correlation between levels of plasma EV LINE‐1 mRNA and plasma brain aging biomarkers.

To investigate the relationship between plasma EV LINE‐1 mRNA levels and aging, the LINE‐1 mRNA levels in EVs isolated from plasma samples of 185 individuals aged 20–95 years were quantified (Figure 1A). Plasma EVs were characterized using NTA, TEM, and Western blotting. NTA confirmed the typical size distribution of EVs (~50–300 nm) (Figure 1B), while TEM revealed their characteristic morphological features (Figure 1C). Western blot analysis validated the presence of EV‐specific markers (ALIX and CD63) and the absence of the negative control markers (GM130 and Calnexin), confirming the purity of the isolated EVs (Figure 1D).

Quantitative PCR (qPCR) using three distinct LINE‐1‐specific primer sets (targeting ORF1, ORF2, and 5′UTR regions, respectively; Table S1) revealed a significant age‐dependent increase in plasma EV LINE‐1 mRNA levels (Figure 1E,G,H). LINE‐1 mRNA levels were the lowest in young individuals (20–45 years; ORF1: 0.6 ± 0.3; ORF2: 0.6 ± 0.3; 5′UTR: 0.4 ± 0.2) and markedly higher in older individuals (> 65 years; ORF1: 7.6 ± 5.0; ORF2: 7.9 ± 5.5; 5′UTR: 6.7 ± 4.4; *p* < 0.001 for all primer sets; Table 1). This increase was consistent across all primer sets, indicating a robust upregulation of LINE‐1 mRNA in plasma EVs with age.

**TABLE 1:** Baseline characteristics of participants in three age groups.

Characteristics	Young (20–45 years; *n* = 58)	Middle‐aged (46–65 years; *n* = 59)	Old (> 65 years; *n* = 68)	*p*‐value
Age (year)	33.7 ± 7.5	56.5 ± 5.1	76.5 ± 7.3	< 0.001
Male, *n* (%)	28 (48.3%)	33 (55.9%)	35 (51.5%)	0.707
Diabetes mellitus, *n* (%)	3 (5.2%)	17 (28.8%)	17 (25.0%)	0.003
Hypertension, *n* (%)	8 (13.8%)	24 (40.7%)	44 (64.7%)	< 0.001
Coronary heart disease, *n* (%)	15 (25.9%)	31 (52.5%)	37 (54.4%)	0.002
SBP (mm Hg)	123.0 ± 15.7	131.8 ± 16.7	143.3 ± 21.0	< 0.001
DBP (mm Hg)	82.9 ± 10.8	84.1 ± 11.6	81.9 ± 10.8	0.526
Heart rate (bpm)	79.8 ± 10.2	78.6 ± 11.7	76.8 ± 12.5	0.353
Currently smoking, *n* (%)	12 (20.7%)	20 (33.9%)	18 (26.5%)	0.272
Currently alcohol drinking, *n* (%)	19 (32.8%)	19 (32.2%)	18 (26.5%)	0.691
ORF1	0.6 ± 0.3	1.9 ± 0.7	7.6 ± 5.0	< 0.001
ORF2	0.6 ± 0.3	2.0 ± 0.7	7.9 ± 5.5	< 0.001
5′UTR	0.4 ± 0.2	1.4 ± 0.5	6.7 ± 4.4	< 0.001
NfL (pg/mL)	4.3 ± 3.2	15.0 ± 10.2	49.3 ± 19.4	< 0.001
Aβ42 (pg/mL)	5.2 ± 3.1	5.3 ± 1.6	6.7 ± 3.7	0.007
pTau181 (pg/mL)	2.8 ± 3.8	3.9 ± 5.8	3.0 ± 1.9	0.29
Aβ40 (pg/mL)	366.5 ± 130.3	394.6 ± 139.4	468.8 ± 167.3	< 0.001
Aβ42/Aβ40 × 100	1.5 ± 0.9	1.5 ± 0.7	1.6 ± 1.1	0.809
Platelets (×109/L)	242.1 ± 51.9	232.6 ± 59.5	235.5 ± 81.5	0.73
HGB (g/L)	135.6 ± 19.3	136.1 ± 16.3	124.6 ± 15.8	< 0.001
RBC (×1012/L)	4.6 ± 0.5	4.5 ± 0.5	4.1 ± 0.5	< 0.001
WBC (×109/L)	6.2 ± 1.7	6.1 ± 1.8	6.2 ± 1.8	0.999
LYMPH% (%)	33.2 ± 8.2	31.2 ± 8.5	29.2 ± 9.3	0.038
LYMPH (×109/L)	2.0 ± 0.6	1.8 ± 0.5	1.7 ± 0.6	0.011
NEUT% (%)	57.9 ± 8.9	59.7 ± 9.3	60.7 ± 10.2	0.258
NEUT (×109/L)	3.6 ± 1.3	3.8 ± 1.7	3.8 ± 1.6	0.788
N/L ratio	1.9 ± 0.9	2.2 ± 1.4	2.7 ± 2.1	0.197
P/L ratio	128.6 ± 40.4	135.2 ± 50.6	155.6 ± 95.1	0.068
HDL‐C (mmol/L)	1.4 ± 0.4	1.2 ± 0.3	1.3 ± 0.3	0.057
LDL‐C (mmol/L)	2.7 ± 0.7	3.0 ± 0.9	2.7 ± 0.9	0.048
CHOL (mmol/L)	4.4 ± 0.9	4.7 ± 1.2	4.4 ± 1.1	0.211
TG (mmol/L)	1.3 ± 0.7	1.5 ± 0.7	1.3 ± 0.6	0.193
APOB (g/L)	0.8 ± 0.3	1.0 ± 0.3	0.9 ± 0.3	0.068
APOA1 (g/L)	1.2 ± 0.3	1.2 ± 0.2	1.2 ± 0.2	0.257
ALB (g/L)	44.7 ± 5.4	42.1 ± 6.3	40.4 ± 7.0	< 0.001
URIC (μmol/L)	329.3 ± 106.3	321.3 ± 84.6	309.4 ± 86.8	0.478
UREA (mmol/L)	5.0 ± 1.4	6.0 ± 1.6	6.0 ± 1.8	< 0.001
HCY (μmol/L)	13.0 ± 6.6	13.9 ± 6.8	16.9 ± 9.1	0.013
GLU (mmol/L)	5.1 ± 1.0	5.8 ± 1.6	5.9 ± 1.9	0.014
AST (U/L)	26.8 ± 37.5	27.7 ± 18.6	23.6 ± 9.3	0.141
ALT (U/L)	29.2 ± 30.4	28.9 ± 24.9	21.6 ± 14.5	0.232
GGT (U/L)	35.8 ± 43.4	28.5 ± 19.1	28.6 ± 17.7	0.863
ALP (U/L)	69.4 ± 19.4	74.4 ± 22.1	79.3 ± 24.7	0.049
GLDH (U/L)	7.9 ± 26.5	4.9 ± 4.5	5.3 ± 6.9	0.509
CREA (μmol/L)	71.0 ± 72.0	62.8 ± 14.8	67.1 ± 18.8	0.529
eGFR	120.1 ± 12.7	105.5 ± 12.3	85.5 ± 17.4	< 0.001

*Note:* Continuous variables are reported as mean ± standard deviation (SD). Group differences are examined using the Kruskal–Wallis *H* test and one‐way ANOVA. *p*‐value indicates the column contains the results of between‐group statistical comparisons, thereby avoiding any potential ambiguity.

Abbreviations: ALB, albumin; ALP, alkaline phosphatase; ALT, alanine aminotransferase; APOA1, apolipoprotein‐A1; APOB, apolipoprotein‐B; AST, aspartate aminotransferase; CHOL, cholesterol; CREA, creatinine; DBP, diastolic blood pressure; eGFR, glomerular filtration rate; GGT, gamma‐glutamyl transpeptidase; GLDH, glutamic acid dehydrogenase; GLU, glucose; HCY, homocysteine; HDL‐C, high‐density lipoprotein cholesterol; HGB, hemoglobin; LDL‐C, low‐density lipoprotein cholesterol; LYMPH%, the percentage of lymphocyte; LYMPH, lymphocyte; N/L ratio, neutrophil to lymphocyte ratio; NEUT%, the percentage of neutrophil; NEUT, neutrophil; P/L ratio, platelets to lymphocyte ratio; RBC, red blood cell; SBP, systolic blood pressure; TG, triglyceride; URIC, uric acid; WBC, white blood cell.

Next, we assessed correlations between plasma EV LINE‐1 mRNA levels and brain aging biomarkers, including neurofilament light chain (NFL), amyloid‐beta 42 (Aβ42), amyloid‐beta 40 (Aβ40), and phosphorylated tau 181 (pTau181). Stratification of participants into tertiles based on LINE‐1 mRNA expression revealed significantly elevated levels of NFL, Aβ42, and Aβ40 in individuals in the highest tertile compared to those in the lowest tertile (Figure 1F,I,K). For example, NFL levels were 4.3 ± 3.2 pg/mL in younger individuals versus 49.3 ± 19.4 pg/mL in older individuals (*p* < 0.001; Table 1). Additionally, Spearman correlation analysis and multivariable regression analysis demonstrated strong positive associations between LINE‐1 mRNA levels and aging biomarkers, particularly NFL (Figure 1J; Table 2). These findings identified plasma EV LINE‐1 mRNA as a non‐invasive marker of aging and brain degeneration.

**TABLE 2:** Multivariable regression analysis of the association between extracellular vesicle (EV) LINE‐1 mRNA level and plasma brain aging biomarkers.

LINE‐1 mRNA level	Model	Statistics	NFL	AΒ42	pTau181	AΒ40	AΒ42/AΒ40 × 100
ORF1	Unadjusted model	*β* (95% CI)	0.17 (0.16, 0.18)	0.43 (0.24, 0.63)	−0.03 (−0.19, 0.12)	0.01 (0.00, 0.01)	0.51 (−0.17, 1.19)
		*p*	< 0.0001	< 0.0001	0.6695	< 0.0001	0.1455
	Model I	*β* (95% CI)	0.17 (0.15, 0.19)	0.13 (0.00, 0.26)	−0.05 (−0.14, 0.05)	0.00 (−0.00, 0.00)	0.19 (−0.24, 0.62)
		*p*	< 0.0001	0.503	0.3109	0.1056	0.3813
	Model II	*β* (95% CI)	0.17 (0.14, 0.20)	0.11 (−0.02, 0.25)	−0.05 (−0.14, 0.05)	0.00 (−0.00, 0.00)	0.27 (−0.15, 0.68)
		*p*	< 0.0001	0.0999	0.3287	0.6514	0.2074
ORF2	Unadjusted model	*β* (95% CI)	0.18 (0.17, 0.19)	0.52 (0.31, 0.73)	−0.04 (−0.21, 0.12)	0.01 (0.01, 0.01)	0.58 (−0.16, 1.31)
		*p*	< 0.0001	< 0.0001	0.6111	< 0.0001	0.1262
	Model I	*β* (95% CI)	0.19 (0.17, 0.22)	0.21 (0.06, 0.35)	−0.06 (−0.16, 0.04)	0.00 (0.00, 0.01)	0.24 (−0.23, 0.71)
		*p*	< 0.0001	0.0051	0.2629	0.0203	0.3208
	Model II	*β* (95% CI)	0.19 (0.16, 0.22)	0.17 (0.02, 0.31)	−0.06 (−0.16, 0.05)	0.00 (−0.00, 0.00)	0.30 (−0.15, 0.75)
		*p*	< 0.0001	0.0262	0.273	0.3034	0.1939
5′UTR	Unadjusted model	*β* (95% CI)	0.15 (0.14, 0.16)	0.40 (0.22, 0.57)	−0.04 (−0.18, 0.10)	0.01 (0.00, 0.01)	0.46 (−0.15, 1.08)
		*p*	< 0.0001	< 0.0001	0.554	< 0.0001	0.1378
	Model I	*β* (95% CI)	0.15 (0.13, 0.17)	0.13 (0.01, 0.25)	−0.06 (−0.14, 0.03)	0.00 (−0.00, 0.00)	0.18 (−0.20, 0.56)
		*p*	< 0.0001	0.0285	0.1971	0.1364	0.357
	Model II	*β* (95% CI)	0.15 (0.12, 0.17)	0.09 (−0.03, 0.20)	−0.05 (−0.13, 0.03)	−0.00 (−0.00, 0.00)	0.23 (−0.13, 0.58)
		*p*	< 0.0001	0.1341	0.2214	0.9386	0.2116

*Note:* Model I is adjusted for age. Model II is adjusted for Model I as well as HGB, RBC, HDL‐C, LDL‐C, TG, APOB, AOPA1, ALB, UREA, HCY, GLU, AST, ALT, ALP, eGFR, SBP, coronary heart disease, diabetes, and hypertension.

### 2.2 Characterization of Plasma EVs Derived From Aged Mice and Its Possible Source

**FIGURE 2:**
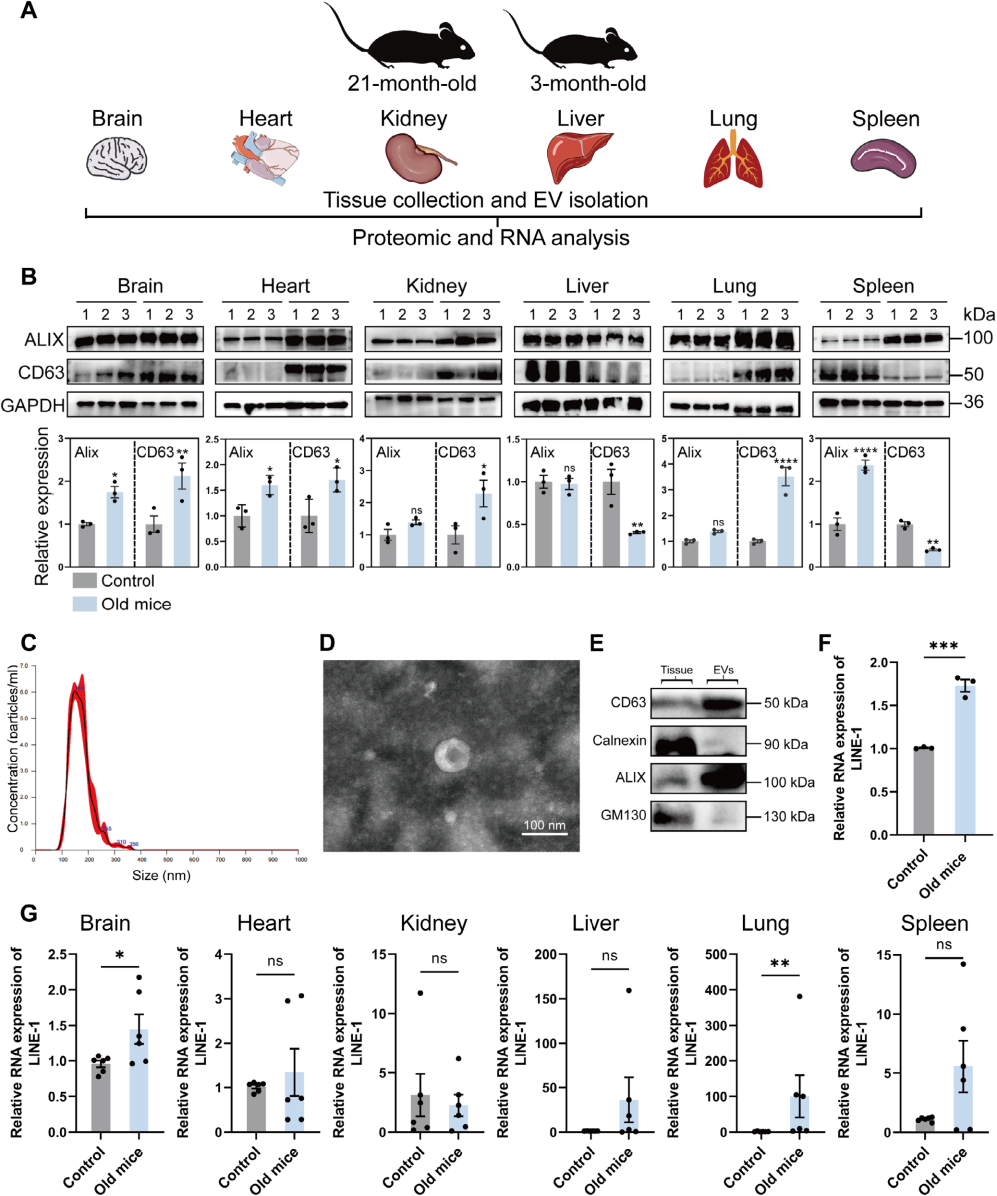
Identifications of extracellular vesicles (EVs) extracted from old mice and its possible source. (A) Schematic representation of the organs collection. (B) Western blot analysis based on tissue lysates from brain, heart, kidney, liver, lung, and spleen of control mice of 3‐month‐old and aged mice of 21‐month‐old (*n* = 3 mice per group). One‐way ANOVA is used to analyze the differences between groups for normally distributed data, and non‐normally distributed data are analyzed by non‐parametric tests. Significant difference from control is determined based on *p < 0.05, **p < 0.01, ***p < 0.001, and ****p < 0.0001, respectively; ns, not significant. (C) Nanoparticle‐tracking analysis (NTA) of plasma EVs extracted from aged mice of 21‐month‐old. (D) Expected size of EVs and morphology based on transmission electron microscopy. (E) Western blotting analysis based on the lysate fractions of mouse tissue and EVs extracted and measured in (C) and (D) using antibodies against the indicated EV markers with GM130 and Calnexin as negative control markers. (F) qPCR analysis of LINE‐1 RNA levels in EVs isolated from plasma of control mice of 3‐month‐old and aged mice of 21‐month‐old (*n* = 3 mice per group). (G) qPCR analysis of LINE‐1 RNA levels in EVs isolated from brain, heart, kidney, liver, lung, and spleen of control mice of 3‐month‐old and aged mice of 21‐month‐old (*n* = 6 mice per group). One‐way ANOVA is used to analyze the differences between groups for normally distributed data, and non‐normally distributed data are analyzed by non‐parametric tests. Significant difference from control is determined based on *p < 0.05, **p < 0.01, ***p < 0.001, and ****p < 0.0001, respectively; ns, not significant.

To investigate the characteristics and tissue origins of plasma EVs in aged mice, organs were collected from 21‐month‐old mice and 3‐month‐old mice (Figure 2A). To determine tissue‐specific contributions to circulating EVs, expression levels of EV‐specific markers (ALIX and CD63) were analyzed in lysates from the brain, heart, kidney, liver, lung, and spleen of aged and young mice (*n* = 3). Western blot results revealed significantly increased ALIX and CD63 levels in the brain and heart of aged mice, compared to controls (*p* < 0.05), whereas no significant differences were observed in the kidney or liver (Figure 2B). These findings suggested that age‐dependent upregulation of EV secretion occurred in specific tissues, particularly the brain and heart, potentially contributing to elevated circulating EV levels in aged mice.

Furthermore, EVs were isolated from plasma of 21‐month‐old WT mice and compared to those of 3‐month‐old controls (*n* = 6). NTA confirmed that the size distribution of aged mouse plasma EVs (~50–300 nm) was consistent with expectation, with a prominent peak at 149 nm (Figure 2C). TEM further revealed the typical morphology of EVs (Figure 2D), and Western blot analysis validated the presence of EV‐specific markers (ALIX and CD63) and the absence of cellular contaminants (GM130 and Calnexin) in aged mouse EVs (Figure 2E). Meanwhile, we also used qPCR to measure the levels of LINE‐1 RNA in plasma EVs from aged and young mice (*n* = 3). The results showed that LINE‐1 RNA expression in plasma EVs from aged mice was significantly elevated compared to young mice, which was consistent with the findings revealed in our human studies (Figure 2F).

Then, to determine tissue‐specific contributions to LINE‐1 RNA in circulating EVs, expression levels of LINE‐1 RNA were analyzed in EVs isolated from the brain, heart, kidney, liver, lung, and spleen of 21‐month‐old and 3‐month‐old mice (*n* = 6; Table S2). qPCR results revealed significantly increased LINE‐1 RNA levels in the brain and lung of aged mice, compared to controls (*p* < 0.05), whereas no significant differences were observed in the heart, kidney, liver, or spleen (Figure 2G). These findings suggested that age‐dependent upregulation of LINE‐1 activities occurred in specific tissues, particularly the brain and lung, potentially contributing to elevated LINE‐1 RNA levels in circulating EVs in aged mice.

### 2.3 Old EVs Can Cause LINE‐1 Associated Cognitive Impairments in Mice

**FIGURE 3:**
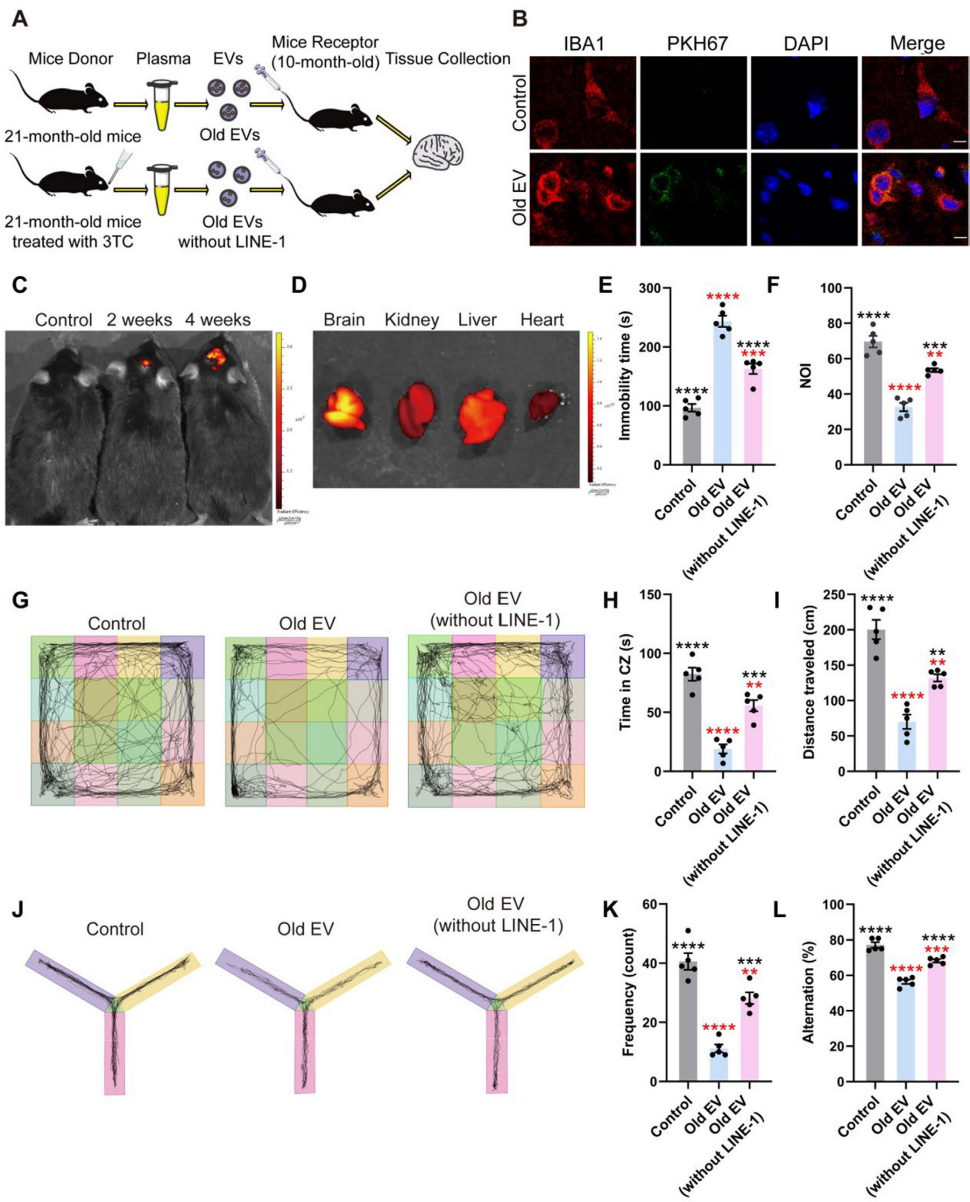
Plasma EVs from old mice cause LINE‐1‐associated behavioral abnormalities in receptor mice (10‐month‐old). (A) Workflow of animal treatment. (B) Representative confocal microscopic images of PKH67‐stained old EVs successfully entering mouse brain by tail vein injection (scale bar = 2.5 μm). (C, D) Representative in vivo images of Dir‐stained old EVs entering and accumulating in mouse brain and major organs. (E) Quantitative analysis of immobility time (s) of mice participating in tail suspension test. (F) The ratio of new object to old object autonomously explored. (G, H, I, K) Path trajectory diagram of the open field experiment and quantitative analysis of residence time (s), total distance (cm), and entry frequency (count) in the central zone of the open field explored by the mice in the open field experiments. (J, L) Path trajectory diagram of the Y maze experiments and percentage of alternations of mice participating in the Y maze experiments (*n* = 5 mice per group). Black asterisks represent statistical differences compared to the Old EV group. Red asterisks represent statistical differences compared to the control group. One‐way ANOVA is used to analyze the differences between groups for normally distributed data, and non‐normally distributed data are analyzed by non‐parametric tests. Significant difference from control is determined based on **p < 0.01, ***p < 0.001, and ****p < 0.0001, respectively.

To examine the functional effects of aged EVs on cognition, EVs from 21‐month‐old mice and 3TC treated 21‐month‐old mice were injected into 10‐month‐old recipient mice via tail vein (Figure 3A). Confocal microscopy of PKH67‐stained EVs confirmed successful entry of old EVs into the brain (Figure 3B), while in vivo imaging of Dir‐labeled EVs revealed their accumulation in the brain and major organs, including the liver, kidney, and heart, over a 4‐week period (Figure 3C,D).

Behavioral tests revealed significant cognitive and exploratory impairments in old EV‐treated mice. In the Y‐maze test, old EV‐treated mice displayed reduced spontaneous alternation percentages, indicative of impaired working memory (Figure 3J,L; *p* < 0.0001). Similarly, new object recognition (NOR) tests revealed reduced exploration ratios in old EV‐treated mice, demonstrating recognition memory deficits (Figure 3F; *p* < 0.0001). In the open field test, old EV‐treated mice exhibited diminished central zone exploration, reduced total distance traveled, and decreased entry frequency into the central zone (Figure 3G–I,K; *p* < 0.0001). Furthermore, prolonged immobility times were observed in the tail suspension test (Figure 3E; *p* < 0.0001), indicative of depressive‐like behavior. Notably, the behavioral performance of the recipient mice injected with EVs extracted from 21‐month‐old mice that were treated with the LINE‐1 inhibitor 3TC were close to near‐control levels across all tests (vs. old EV group; *p* < 0.01). These results demonstrated that old EVs impaired cognition through mechanisms linked to LINE‐1 activity.

### 2.4 Old EVs Can Induce LINE‐1 Associated Brain Aging

**FIGURE 4:**
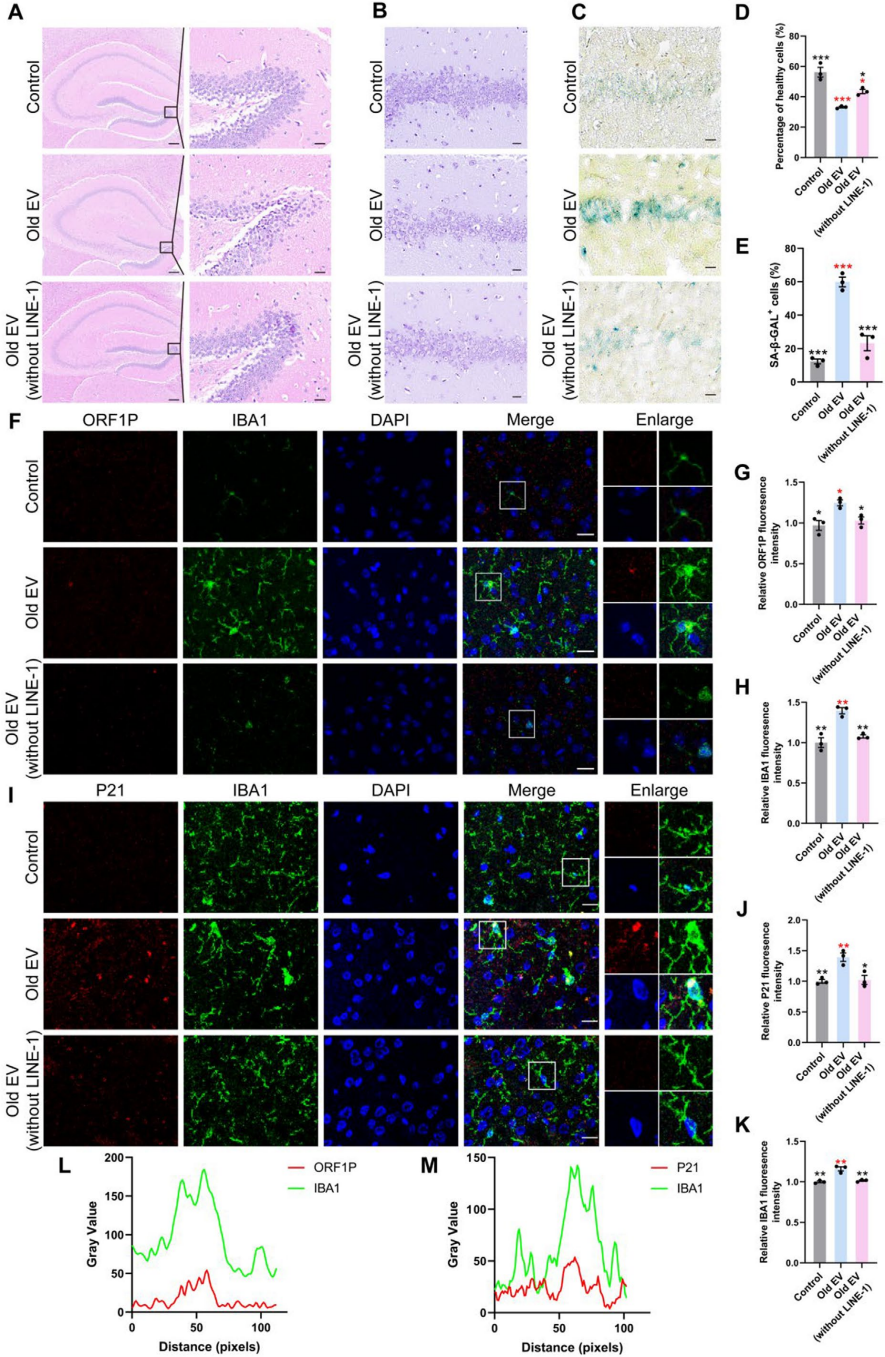
LINE‐1 in EVs targets microglia to cause premature brain aging in mice. (A) Photomicrographs of H&E‐stained hippocampus sections for each group of mice (left: Scale bar = 100 μm; right: Scale bar = 20 μm). (B) Identification of neuronal survival base on Nissl staining in the hippocampus (scale bar = 20 μm). (C) Photomicrographs of SA‐β‐gal stained hippocampus sections for each group of mice (scale bar = 20 μm). (D) Percentage of healthy neuronal cells in each visual field with densely stained Nissl bodies (*n* = 3). (E) Percentage of positive SA‐β‐gal cells in each visual field. (F) Fluorescence co‐localization of IBA1 (green), ORF1P (red), and DAPI (blue) in the mouse brain (scale bar = 20 μm). (G, H) Quantitative analysis of IBA1‐positive cells and ORF1P expression in the mouse brain. (I) Fluorescence co‐localization of IBA1 (green), P21 (red), and DAPI (blue) in the mouse brain (scale bar = 20 μm). (J, K) Quantitative analysis of IBA1‐positive cells and P21 expression in the mouse brain. Black asterisks represent statistical differences compared to the Old EV group. Red asterisks represent statistical differences compared to the control group. One‐way ANOVA is used to analyze the differences between groups for normally distributed data, and non‐normally distributed data are analyzed by non‐parametric tests. Significant different from control is determined based on *p < 0.05, **p < 0.01, and ***p < 0.001, respectively. (L) Colocation analysis of ORF1P (red) and IBA1 (green) in (F). (M) Colocation analysis of P21 (red) and IBA1 (green) in (I).

Histological analysis of hippocampal sections revealed significant structural damage, reduced neuronal density, and disrupted cellular architecture in old EV‐treated mice, as observed by H&E staining (Figure 4A). Nissl staining further confirmed a reduction in the number of surviving neurons in old EV‐treated mice, which was significantly more in old EV (without LINE‐1)‐treated mice (*p* < 0.05) (Figure 4B,D). As shown by SA‐β‐gal staining, old EV‐treated mice exhibited a significant increase in SA‐β‐gal activity in the hippocampus sections (*p* < 0.001) (Figure 4C,E). Microglial activation, assessed by IBA1 immunofluorescence, was significantly increased in old EV‐treated mice (Figure 4H,K), showing morphological changes, for example, enlarged soma and decreased number of branches (Figure S1A,B). Compared to old EV‐treated mice, old EV (without LINE‐1)‐treated mice showed restored neuronal density and reduced microglial activation (*p* < 0.05).

LINE‐1 ORF1P and P21 expression was enriched in IBA1‐positive microglial cells in old EV‐treated mice, linking LINE‐1 RNA to microglial activation and senescence (Figure 4L,M). Old EV (without LINE‐1)–treated mice showed decreased expression of ORF1P and P21 and reduced microglial activation and senescence (Figure 4F,G,I,J). These findings suggested that old EVs promoted brain aging through LINE‐1 RNA‐mediated microglial activation, which could be mitigated by LINE‐1 inhibition.

### 2.5 LINE‐1 RNA Activates the cGAS/STING Pathway to Drive Neuroinflammation

**FIGURE 5:**
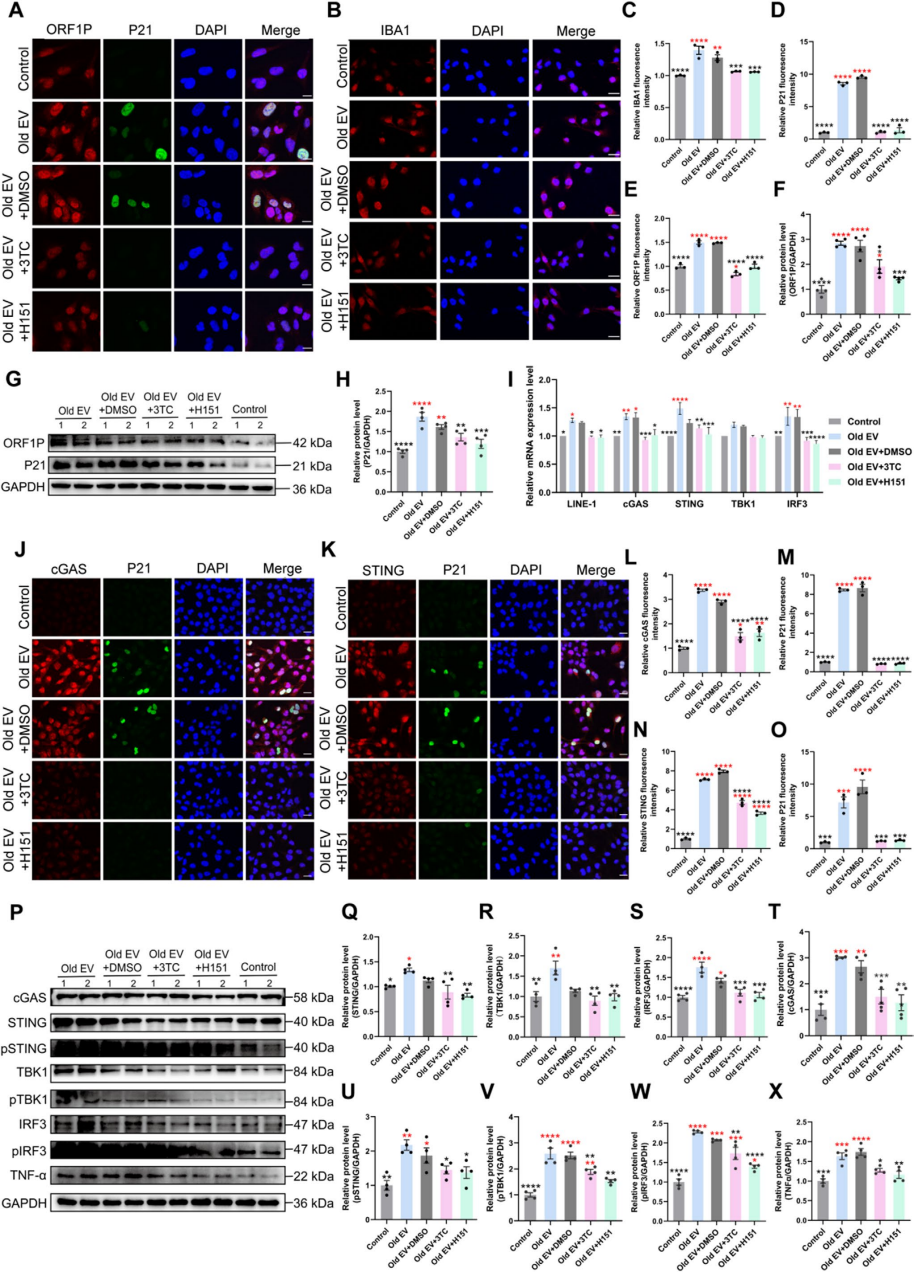
Inhibition of LINE‐1 reverse transcription or the cGAS/STING pathway can ameliorate the old EV‐induced microglia senescence. (A) Representative images of immunofluorescence detection of LINE‐1 ORF1P and P21 in microglia (scale bar = 20 μm). (B, C) Representative images and quantification of microglia activation revealed by immunostaining with anti‐IBA‐1 antibody (scale bar = 40 μm). (D, E) Quantification of immunofluorescence analysis in (A). (G) Western blot analysis of LINE‐1 ORF1P and P21. (F, H) Quantification of Western blot analysis presented in (G). (I) Gene expression of *LINE‐1*, *cGAS*, *STING*, *TBK1*, and *IRF3* based on real‐time quantitative PCR. (J) Representative images of immunofluorescence detection of cGAS and P21 in microglia (scale bar = 40 μm). (L, M) Quantification of immunofluorescence analysis presented in (J). (K) Representative images of immunofluorescence detection of STING and P21 in microglia (scale bar = 40 μm). (N, O) Quantification of immunofluorescence analysis presented in (K). (P) Western blot analysis of cGAS, STING, pSTING, TBK1, pTBK1, IRF3, pIRF3, and TNF‐α. (Q–X) Quantification of Western blot analysis presented in (P). Black asterisks represent statistical differences compared to the Old EV group. Red asterisks represent statistical differences compared to the control group. One‐way ANOVA is used to analyze the differences between groups for normally distributed data, and non‐normally distributed data are analyzed by non‐parametric tests. Significant difference from control is determined based on *p < 0.05, **p < 0.01, ***p < 0.001, and ****p < 0.0001, respectively.

Immunofluorescence and Western blot analysis revealed significant activation of microglia and the cGAS/STING pathway in old EV‐treated HMC3 (Figure 5B,C), as indicated by elevated expression of cGAS, STING, phosphorylated TBK1 (pTBK1), and phosphorylated IRF3 (pIRF3) (Figure 5I,P,Q–X; *p* < 0.001). Co‐localization studies confirmed the enrichment of ORF1P, cGAS, STING, and P21 in microglia, highlighting the linkage between cGAS/STING activation and cellular senescence (Figure 5A,D–H). Both 3TC and STING inhibitor H151 suppressed cGAS/STING signaling and reduced downstream inflammation. These findings demonstrated that LINE‐1 RNA in plasma EVs promoted neuroinflammation through activation of microglia and the cGAS/STING pathway, which could be targeted pharmacologically to mitigate aging‐associated brain pathology.

### 2.6 Inhibition of LINE‐1 Activity Improves Cognitive Function via the cGAS/STING Pathway

**FIGURE 6:**
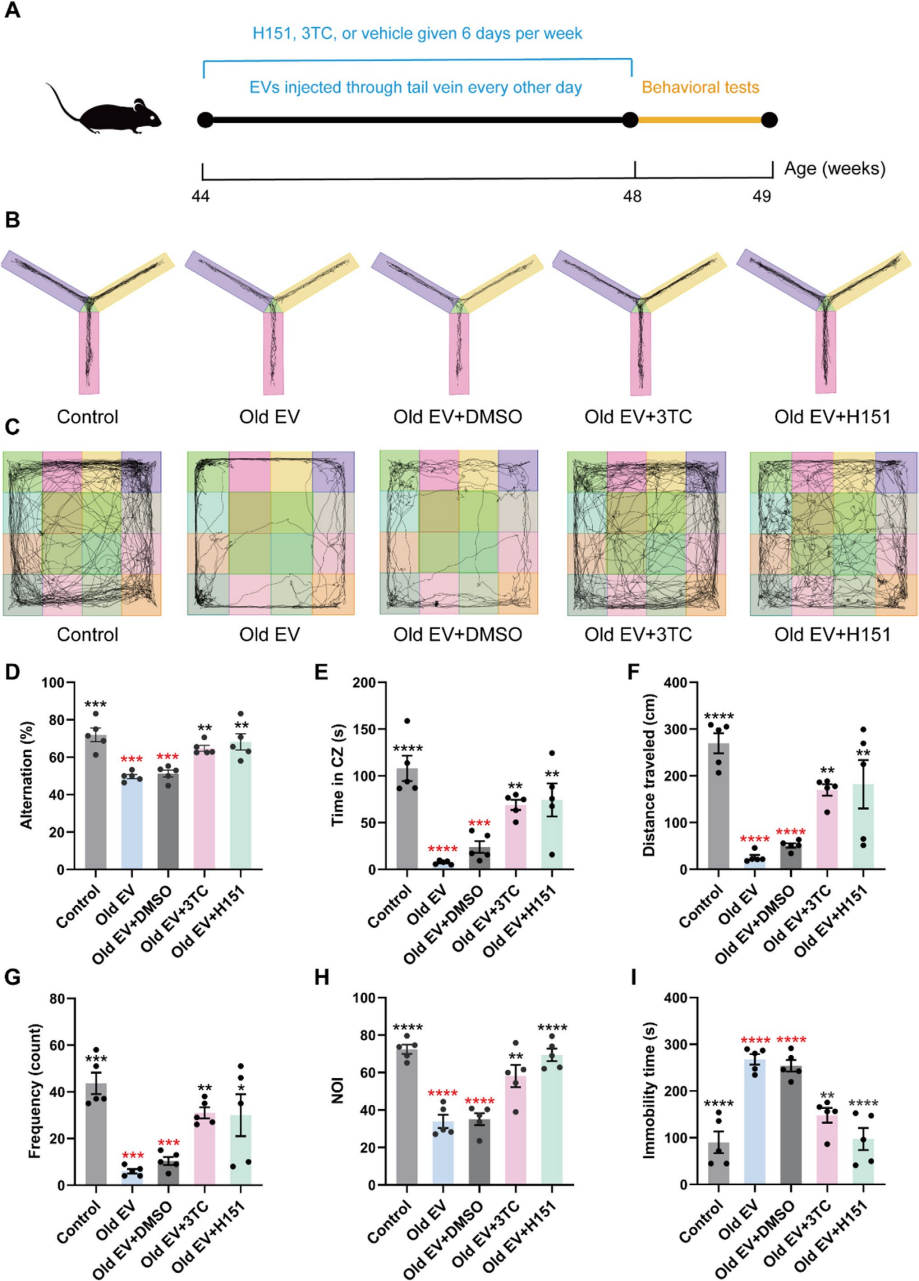
Inhibition of LINE‐1 reverse transcription or the cGAS/STING pathway can ameliorate the old EV‐induced behavioral abnormalities. (A) Schematic presentation of the animal experiment procedures. (B) Path trajectory diagram of the Y maze experiments. (C) Path trajectory diagram of the open field experiment. (D) Percentage of alternations of mice participating in the Y maze experiments. (E, F, G) Quantitative analysis of residence time (s), total distance (cm), and entry frequency (count) in the central area explored by the mice in the open field experiments. (H) Ratio of new object to old object autonomously explored. (I) Immobility time (s) of mice participating in tail suspension experiment (*n* = 5 mice per group). Black asterisks represent statistical differences compared to the Old EV group. Red asterisks represent statistical differences compared the control group. One‐way ANOVA is used to analyze the differences between groups for normally distributed data, and non‐normally distributed data are analyzed by non‐parametric tests. Significant difference from control is determined based on *p < 0.05, **p < 0.01, ***p < 0.001, and ****p < 0.0001, respectively.

To test whether inhibiting LINE‐1 activity or the cGAS/STING pathway could rescue cognitive impairments induced by old EVs, mice injected with old EVs were treated with either the LINE‐1 reverse transcriptase inhibitor 3TC or the STING inhibitor H151. The experimental timeline was illustrated in Figure 6A. Behavioral assessments showed significant improvements in cognitive and exploratory behaviors in both treatment groups.

In the Y‐maze test, old EV‐treated mice exhibited a significant reduction in spontaneous alternation percentages (*p* < 0.001), indicating impaired working memory (Figure 6B,D). Both 3TC and H151 treatments restored alternation percentages to levels comparable to that of the old EV group (*p* < 0.01). Similarly, in the open field test, old EV‐treated mice showed reduced central zone (CZ) residence time, total distance traveled, and entry frequency, which were consistent with diminished exploratory activity (Figure 6C,E–G; *p* < 0.001). Both inhibitors significantly rescued these impairments (*p* < 0.05), bringing behavioral metrics close to control levels.

In the NOR test, old EV‐treated mice demonstrated reduced exploration of the novel object, as observed by a lower new‐to‐old object exploration ratio (*p* < 0.0001; Figure 6H). Treatment with 3TC or H151 restored recognition memory, with exploration ratios significantly increased compared to untreated old EV groups (*p* < 0.01). Furthermore, in TST, old EV‐treated mice displayed prolonged immobility times, indicative of depressive‐like behavior (Figure 6I; *p* < 0.001). Both inhibitors significantly reduced immobility times, restoring affective behavior to near‐control levels (*p* < 0.01).

These results demonstrated that inhibition of LINE‐1 activity with 3TC or suppression of the cGAS/STING pathway with H151 effectively mitigated cognitive, exploratory, and affective deficits induced by old EVs. These findings suggested the therapeutic potential of targeting the LINE‐1/cGAS/STING axis to preserve brain function during aging.

### 2.7 Inhibition of LINE‐1 Activity Ameliorates Pathological Changes via the cGAS/STING Pathway

**FIGURE 7:**
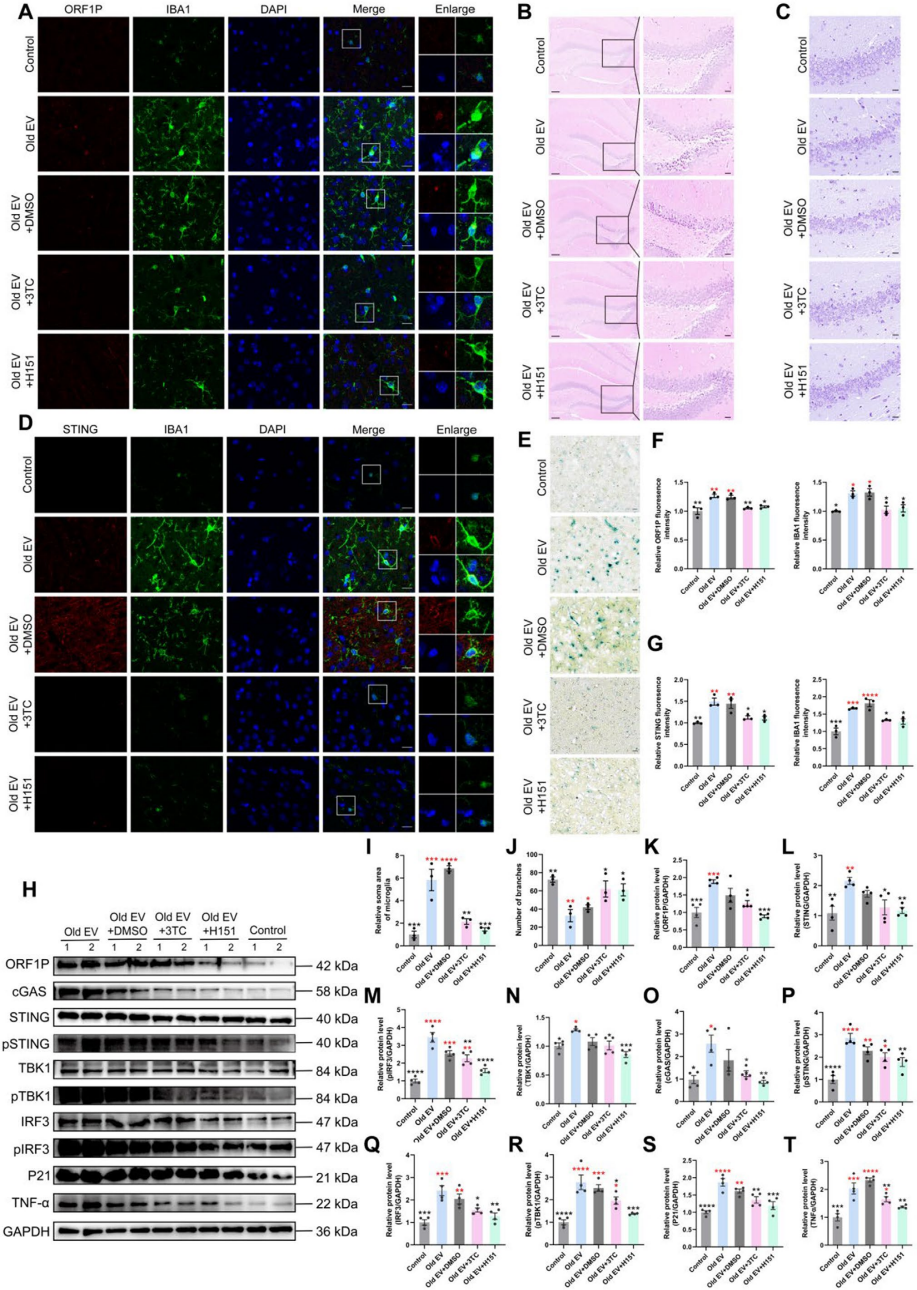
Inhibition of LINE‐1 reverse transcription or the cGAS/STING pathway can ameliorate the old EV‐induced premature brain aging. (A) Representative images of immunofluorescence detection of LINE‐1 ORF1P and IBA1 in the mouse brain (scale bar = 20 μm). (B) Photomicrographs of H&E‐stained hippocampus sections for each group of mice (left: Scale bar = 100 μm; right: Scale bar = 20 μm). (C) Identification of neuronal survival by Nissl staining in the hippocampus (scale bar = 20 μm). (D) Representative images of immunofluorescence detection of STING and IBA1 in the mouse brain. (E) Photomicrographs of SA‐β‐gal stained hippocampus sections for each group of mice (scale bar = 20 μm). (F) Quantification of immunofluorescence analysis presented in (A). (G) Quantification of immunofluorescence analysis presented in (D). (H) Western blot analysis based on LINE‐1 ORF1P, cGAS, STING, pSTING, TBK1, pTBK1, IRF3, pIRF3, P21, and TNF‐α. (I, J) Microglia morphological analysis. (K–T) Quantification of Western blot analysis presented in (I). Black asterisks represent statistical differences compared to the Old EV group. Red asterisks represent statistical differences compared to the control group. One‐way ANOVA is used to analyze the differences between groups for normally distributed data, and non‐normally distributed data are analyzed by non‐parametric tests. Significant difference from control is determined based on *p < 0.05, **p < 0.01, ***p < 0.001, and ****p < 0.0001, respectively.

To further explore the mechanistic basis of the pathological effects of old EVs on the brain, hippocampal sections from mice treated with old EVs, with or without 3TC or H151, were analyzed. Immunofluorescence analysis revealed elevated LINE‐1 ORF1P and IBA1 expression in old EV‐treated mice, consistent with increased LINE‐1 activity and microglial activation (Figure 7A,F,G, S2B; *p* < 0.05). Both 3TC and H151 treatments significantly reduced these markers (*p* < 0.05), indicating successful suppression of LINE‐1 activity and microglial activation.

Histological analysis of hippocampal sections using H&E staining showed disrupted neuronal architecture, reduced neuronal density, and structural abnormalities in the old EV group (Figure 7B). Nissl staining confirmed a significant reduction in healthy neurons in old EV‐treated mice compared to controls (*p* < 0.001; Figure 7C). SA‐β‐gal staining also showed that old EV‐treated mice exhibited a significant increase in SA‐β‐gal activity compared to controls (*p* < 0.001) (Figure 7E and Figure S2A). Both 3TC and H151 treatments ameliorated cellular senescence, restored neuronal density, and preserved hippocampal structure.

Co‐localization studies of STING and IBA1 revealed increased STING expression in activated microglia of old EV‐treated mice (Figure 7D,G and Figure S2C; *p* < 0.01). Western blot analysis confirmed the activation of the cGAS/STING pathway, with elevated expression of cGAS, STING, phosphorylated STING (pSTING), TBK1, phosphorylated TBK1 (pTBK1), IRF3, phosphorylated IRF3 (pIRF3), and the pro‐inflammatory cytokine TNF‐α in the old EV group (*p* < 0.05; Figure 7H,K–T). Both 3TC and H151 significantly suppressed the activation of these signaling molecules, reducing neuroinflammation (*p* < 0.01). Microglial morphology analysis revealed increased soma size and reduced branching in old EV‐treated mice, indicative of a pro‐inflammatory state (*p* < 0.01; Figure 7I,J). Both treatments restored microglial morphology to quiescent states, further confirming their anti‐inflammatory effects (*p* < 0.05).

Building upon these findings, we further investigated whether similar alterations occurred in neuronal populations across the brain. Immunofluorescence analysis and co‐localization studies of LINE‐1 ORF1P, STING, P21, and NeuN revealed elevated expression of LINE‐1 ORF1P, STING, and P21 in activated neurons in the cortical sections of the old EV‐treated mice (Figures S3 and S4A,C,D,F). Both 3TC and H151 treatments significantly reduced these markers (*p* < 0.05), indicating successful suppression of LINE‐1 activity, cGAS/STING pathway activation, and neuron senescence, which is consistent with the microglial expression patterns observed in immunofluorescence and Western blot analyses. Nissl staining also confirmed a significant reduction in healthy neurons in the cortical sections of old EV‐treated mice (Figure S4B,E).

These findings established that LINE‐1 RNA drove pathological changes in the brain via the cGAS/STING pathway, promoting microglial activation and neuroinflammation. Inhibition of either LINE‐1 activity or cGAS/STING signaling ameliorated these pathological changes, highlighting the potential of these pathways as therapeutic targets.

## 3 Discussion

**FIGURE 8:**
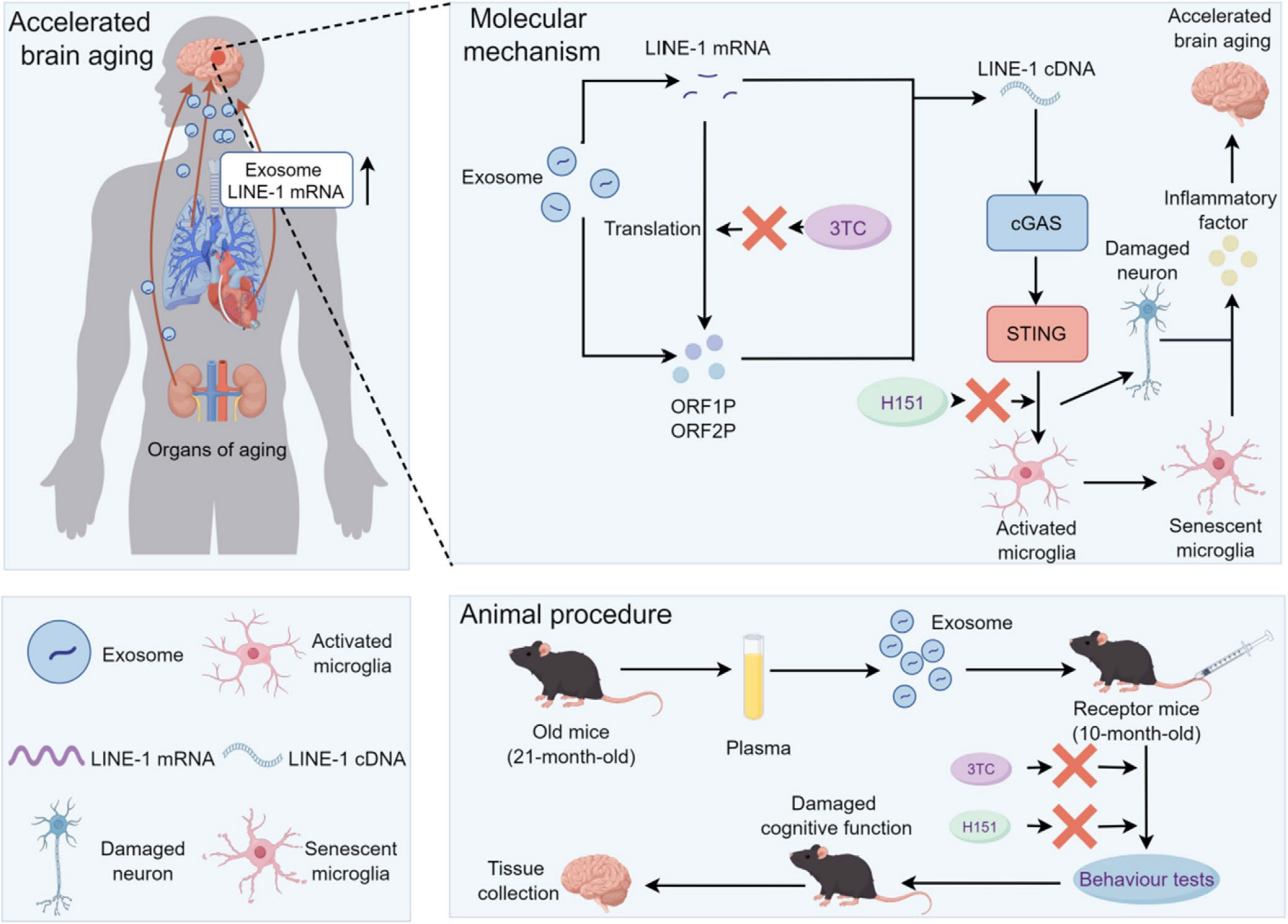
Plasma exosome‐derived LINE‐1 RNA activates the cGAS‐STING pathway and causes brain aging. 3TC inhibits the reverse transcriptase to generate LINE‐1 cDNA that can be sensed by cGAS to activate microglia and initiate neuroinflammation through STING, ultimately causing neuron damage and accelerating brain aging. H151 suppresses neuroinflammation by binding to STING and inhibits STING‐mediated activity. The Figure was created by Figdraw (www.figdraw.com).

This study uncovers a novel and systemic mechanism linking peripheral aging processes to brain dysfunction, highlighting plasma EV‐derived LINE‐1 RNA as a key driver of neuroinflammation and cognitive decline through activation of the cGAS‐STING pathway (Figure 8). Importantly, our findings demonstrate that LINE‐1 associated EVs originated from peripheral aging organs, such as heart and lung, and act as molecular messengers that cross the BBB and propagate aging signals to the brain. This systemic dissemination of pro‐aging factors offers a paradigm shift in understanding brain aging, positioning peripheral tissues as active contributors rather than passive bystanders in neurodegeneration.

The ability of EVs to transport LINE‐1 RNA into the brain introduces a novel layer of complexity to aging‐associated neuroinflammation. Once internalized by microglia, the resident immune cells of the brain (Hanamsagar et al. 2017), LINE‐1 RNA exhibits transcriptional activity, leading to the production of LINE‐1 proteins (ORF1P and ORF2P) or reverse transcription into LINE‐1 cDNA (Roy et al. 2024). This process subsequently activates the innate immune sensor cGAS, triggering downstream STING signaling, cytokine release, microglial senescence, and neuronal damage (Gamdzyk et al. 2020; Tang et al. 2024). This mechanistic pathway identifies EV‐derived LINE‐1 RNA as a previously unrecognized factor contributing to age‐related brain pathology. Furthermore, the transcriptional activity of LINE‐1 RNA within recipient cells amplifies its downstream effects, perpetuating neuroinflammation and cellular dysfunction in a feed‐forward loop.

Our findings extend the role of LINE‐1 activity beyond its established involvement in genomic instability and cellular stress (Saleh et al. 2019). While prior studies primarily focused on intracellular LINE‐1 mobilization (Kirilyuk et al. 2008), our study demonstrates that LINE‐1 RNA can be exported via EVs, enabling systemic dissemination and intercellular transfer. This process effectively propagates aging signals across tissues, reinforcing the interconnected nature of systemic and brain aging. This systemic aging signal transmission pattern provides a new perspective for understanding the synergistic development mechanisms of multi‐organ aging. The transcriptional activity of LINE‐1 RNA in recipient cells also provides evidence to support that LINE‐1 elements can act as mobile genetic elements, amplifying their effects through secondary transcription and protein production. This property likely contributes to both local (brain‐specific) and systemic inflammatory responses, implicating LINE‐1 RNA in broader aging‐related dysfunction beyond the brain.

Microglia emerge as key mediators of this inflammatory cascade. In their normal state, microglia maintain brain homeostasis by clearing cellular debris and responding to injury (Gomez‐Nicola and Perry 2015; Umpierre and Wu 2021). However, aging induces a shift toward a pro‐inflammatory, senescent phenotype, as evidenced by increased expression of activation markers, such as IBA1, and morphological changes, including larger soma size and reduced branching complexity (Bowyer et al. 2020). Our study demonstrates that EV‐derived LINE‐1 RNA directly accelerates this transition, activating microglia through the cGAS‐STING pathway and exacerbating inflammation and neurotoxicity. This microglial dysfunction likely underpins the observed neuronal damage, cognitive impairments, and depressive‐like behaviors in old EV‐treated mice. Importantly, pharmacological inhibition of LINE‐1 activity (via 3TC) or STING signaling (via H151) effectively restored microglial homeostasis, suppressed inflammation, and improved behavioral outcomes, highlighting these pathways as promising therapeutic targets (Gulen et al. 2023; Li et al. 2021).

The systemic origin of EV‐derived LINE‐1 RNA also suggests broader implications for aging and age‐related diseases. Aging organs likely act as sources of inflammatory EVs, contributing to systemic aging phenotypes that extend beyond the brain. Our study revealed increased EV production and LINE‐1 RNA expression in peripheral tissues, including the heart and lung, suggesting that systemic inflammation and tissue dysfunction were likely interconnected through EV‐mediated signaling. Moving forward, a key priority will be to determine the precise tissue or cellular sources of these circulating EVs in vivo. Future studies employing techniques like immunocapture with tissue‐specific markers are necessary to map their dissemination.

This also highlights the need for future studies to investigate the effects of LINE‐1 RNA on non‐neuronal tissues and to evaluate whether modulating EV biogenesis or cargo selection could suppress aging signals and mitigate tissue‐specific aging phenotypes.

Another critical finding of this study is the transcriptional activity of EV‐delivered LINE‐1 RNA in recipient cells. This observation raises the intriguing possibility that LINE‐1 RNA serves not only as a signaling molecule but also as a template for amplifying cellular stress responses and genomic instability. This amplification mechanism may underlie the persistence of inflammatory states and cellular senescence, particularly in the brain. Future research should explore whether similar transcriptional effects occur in peripheral tissues and assess their contribution to systemic inflammation and metabolic dysfunction in aging.

Therapeutically, our findings establish the feasibility of targeting both LINE‐1 RNA activity and its downstream signaling pathways to counteract age‐related dysfunction. The ability of 3TC to inhibit LINE‐1 reverse transcription effectively prevented cGAS activation, while H151 suppressed STING‐mediated inflammation, collectively preserving cognitive function and reducing neuroinflammation. These dual approaches highlight distinct but complementary strategies for intervention—targeting the upstream source (LINE‐1 RNA) and downstream effectors (STING signaling). Given the systemic nature of EVs, such treatments may also extend to peripheral aging processes, presenting broader applications for age‐related diseases, including metabolic disorders, cardiovascular diseases, and cancers.

Our findings add to a growing body of evidence indicating that EVs in circulation can exert biologically significant effects on the brain. For instance, a 2024 study published in *Nature Aging* demonstrated that plasma EVs from young mice can reverse age‐related functional declines in aged mice by improving mitochondrial energy metabolism (Chen et al. 2024). Additionally, neuronal‐derived EVs from individuals with Down syndrome have been reported to propagate tau pathology in wild‐type mice (Ledreux et al. 2021). Together, these observations indicate that EV‐mediated signaling may link peripheral aging and systemic inflammation to brain health, underscoring the importance of understanding how circulating EV cargo influences long‐term brain homeostasis in aging and disease. This further suggests that in clinical settings involving the transfer of blood components, EVs may influence long‐term neurological outcomes, especially in elderly or vulnerable recipients. Future studies are needed to clarify the role of transfusion‐associated EVs in the central nervous system and their clinical implications.

While this study provides mechanistic insights into the role of EV‐derived LINE‐1 RNA in brain aging, several questions remain unanswered. First, whether EV‐mediated LINE‐1 signaling affects other cell types, such as astrocytes or neurons, warrants further investigation. Second, the long‐term impact of targeting LINE‐1 or cGAS‐STING pathways on systemic aging and neurodegenerative diseases, such as AD and PD, should be explored. Third, understanding how EV cargo is selectively packaged and whether this process can be therapeutically manipulated is crucial for developing targeted interventions.

In conclusion, this study identifies EV‐derived LINE‐1 RNA as a central mediator of brain aging, providing mechanistic evidence to support that the EV‐derived LINE‐1 RNA activates the cGAS‐STING pathway to drive neuroinflammation and cognitive decline (Figure 8). By demonstrating that pharmacological inhibition of LINE‐1 activity and STING signaling effectively ameliorates these effects, our study highlights new avenues for therapeutic intervention. Moreover, the systemic nature of EV signaling underscores the interconnectedness of aging processes across tissues, suggesting that targeting EV cargo may have far‐reaching implications for combating both brain‐specific and systemic aging phenotypes.

## 4 Experimental Procedures

### 4.1 Clinical Study

The Ethics Committee for Biomedical Research Involving Human Beings of Provincial Hospital affiliated to Shandong First Medical University approved the human studies. Informed consent was obtained from all participants. A total of 185 individuals were chosen for this study, excluding those who were diagnosed with cancer, HIV, any kind of brain disease, or tested positive for the hepatitis C or B surface antigen antibodies, and divided into three age groups: young (20–45 years old), middle‐aged (46–65 years old), and old (66–95 years old). Smoking and alcohol use were identified as either current users or non‐users. The demographics of the participants were provided in Table 1. Blood sample from each participant was collected, kept in EDTA anticoagulant‐coated tubes, and processed within 4 h of hospitalization. Plasma was obtained by centrifuging blood samples at 3000 rpm for 15 min, and then divided into small portions (500 μL) and stored at −80°C for later use. For the measurement of brain aging markers, the frozen plasma was melted at 4°C and centrifuged at 10,000 × *g* for 10 min to remove cell debris. Then, the plasma sample was divided into small portions (75 μL) to measure the levels of brain aging biomarkers using Single‐Molecule Analyzer (AXL‐2000, Lychix, Suzhou, China).

### 4.2 Experimental Animals

The 10‐ and 21‐month‐old wild‐type (WT) C57BL/6J mice were purchased from Jinan Xingkang Bio‐tech (Jinan, China) and housed in a temperature‐controlled animal facility with a 12‐h/12‐h cycle of light/darkness and free access to sterile water and food. One week was given for the mice to adapt to the environment before any treatments. For donor mice, the 21‐month‐old mice were selected into two groups: one group received oral 3TC at a dose of 100 mg/kg per day 6 day a week for 4 weeks, and the other received an equivalent volume of PBS for the same duration. Then the mice were euthanized by inhalation of isoflurane. Blood was collected in EDTA anticoagulant‐coated tubes via cardiac puncture. Then, brain, kidney, liver, lung, spleen, and heart tissues of mice were collected. Plasma was obtained by centrifuging blood samples at 3000 rpm for 15 min and then stored at −80°C prior to use.

### 4.3 Isolation and In Vivo Tracking of EVs

Mice or human plasma EVs were extracted from 500 μL of plasma using the Total Exosome Isolation Kit (UR52151, Umibio Biotechnology, Shanghai, China) according to the manufacturer's instructions. The EVs from various organ tissues of 21‐month‐old and 3‐month‐old mice were extracted from 0.1 g of the corresponding organ tissues using the Exosome Isolation and Purification Kit (UR52160, Umibio Biotechnology, Shanghai, China) according to the manufacturer's instructions. For the in vivo tracking of EV distribution, EVs were stained with Dir (UR21017, Umibio Biotechnology, Shanghai, China) and PKH67 (UR52303, Umibio Biotechnology, Shanghai, China), respectively. For Dir staining, EVs were incubated with Dir dye in the dark at 37°C for 30 min. For PKH67 staining, PKH67 dye was added into EVs, and the mixture was shaken for 1 min and then incubated in the dark at 37°C for 10 min. Both of the reactions were terminated by the addition of an equal volume of 1% BSA dissolved in PBS. The samples were then processed using the EV isolation method to remove excess dye, and 200 μg of Dir‐stained EVs and PKH67‐stained EVs were injected into the tail vein of recipient mice, respectively, with the same volume PBS injected into the tail veins of control mice. The mice that were injected with PKH67‐stained EVs were euthanized by inhalation of isoflurane 4 h after the injection, and the brain was collected. The Dir‐stained EVs were injected to the mice every other day for 4 weeks, and then the mice were eventually euthanized by inhalation of isoflurane. Brain, kidney, liver, and heart tissues were collected and imaged using an IVIS spectrum in vivo imaging system (IVIS Lumina II, PerkinElmer).

### 4.4 EV Characteristics

Freshly isolated human or mice plasma EVs were allowed to adsorb onto formvar/carbon coated grids, rinsed briefly in water, and negatively stained in 2% aqueous uranyl acetate. Then, the samples were observed with a transmission electron microscope (TEM, Hitachi H7700, Japan) at 100 kV.

Filtered PBS was used to dilute isolated EV samples from mouse plasma by a factor of 35. The mouse plasma EV size distribution and concentration were examined with nanoparticle tracking analysis (NTA) on a NanoSight NS300 (Malvern Instruments Ltd., Malvern, UK). Samples were recorded at camera level of 11 and detect threshold of 5 using NTA 3.4 Build 3.4.4 software. The human plasma EVs were observed on Particle Metrix (PMX, German) using ZetaView version 8.05.14 SP7.

### 4.5 Animal Treatment

The 3TC (HY‐BO250, USA) and H151 (HY‐112693, USA) were purchased from MCE (USA). On the day of each experiment, the drugs were freshly prepared. Old EVs extracted from the 21‐month‐old mice were quantified using the BCA Protein Analysis Kit (PC0020, Solarbio, Beijing, China). The recipient mice (44‐week‐old) were first randomly divided into three groups (*n* = 5 mice per group): a test group injected with 200 μg protein equivalent of plasma EVs extracted from 21‐month‐old mice into the tail vein every other day for 4 weeks, a drug treatment group injected with 200 μg protein equivalent of plasma EVs extracted from 21‐month‐old mice treated with 3TC into the tail vein every other day for 4 weeks, and a control group injected with an equal amount of PBS for the same duration.

After the findings that Old EVs could induce cognitive impairments, the mechanisms underlying this induction were further explored. The mice were randomly divided into five groups (*n* = 5 mice per group), four of which were injected with 200 μg protein equivalent plasma EVs extracted from 21‐month‐old mice into the tail vein every other day for 4 weeks, and one group was injected with an equal amount of PBS in the tail vein for the same duration as a control. Among the four groups injected with 21‐month‐old mice EVs, one group remained untreated, one group received oral 3TC at a dose of 100 mg/kg per day 6 day a week for 4 weeks, one group was injected intraperitoneally with 750 nmol H‐151 in 200 μL PBS of 5% Tween‐80 and 5% DMSO 6 day a week for 4 weeks, and one group was injected intraperitoneally with vehicle as control. All experimental procedures were authorized by Shandong Provincial Hospital's Laboratory Animal Management and Ethics Review Committee (NSFC: No. 2022–511) and complied with the National Institutes of Health's (NIH, USA) Guide for the Care and Use of Laboratory Animals.

### 4.6 Y‐Maze Test

Through the detection of spontaneous alternating behavior in mice, the Y‐maze is a classic experiment used to test short‐term memory ability. In the maze facility, each mouse was positioned at the end of an arm of the maze. The order in which the mice entered and exited each arm over a period of 5 min was recorded and analyzed. Three consecutive entries into three different arms were considered correct choices. The number of correct choices and the total number of arms entered were recorded for each mouse, and the percentage of alternation was calculated using the following formula: alternation percentage (%) = [(number of correct choices)/(total number of arms entered − 2)] × 100%. The maze facility was wiped with alcohol after each mouse finished its test.

### 4.7 Open Field Test

The anxiety behaviors of mice were evaluated using the open field test, the classic experimental method for evaluating the anxiety behaviors of mice. The facility was a cubic cage with 50 cm high walls, enclosing a 56 cm × 56 cm wooden floor. Each mouse was placed in the corner of the open field at the beginning of each experiment and given 5 min to roam around. The time spent in the central zone (26 × 26 cm), the total traveled distance, and the number of entries in the central region were all recorded using video tracking software. The facility was cleaned with alcohol after each mouse finished its test.

### 4.8 New Object Recognition

Recollection memory of mice was evaluated using the new object recognition test. The open field test was carried out the day before the new object recognition test to serve as the adaptation phase of the new object recognition test (Day 1). On Day 2, mice were given 10 min to explore the same field with two identical objects. Then, the mice were put back into their cages for 2 h. Then, mice were placed in their designated locations for the last time, which now included one familiar object and one new object. They were given 5 min to explore at their leisure. The analyses included mice that investigated the study object for at least 10 s. The amount of time spent with each object was calculated by analyzing the full 5‐min video. The score was calculated using the following formula: new object recognition index (NOI) = (TN − TF)/(TN + TF), where TN was the amount of time spent on the new object and TF was the amount of time spent on investigating the familiar object.

### 4.9 Tail Suspension Test

The depression level in mice was evaluated using the traditional tail suspension test (TST). The mice were placed in a 40 cm high white open box and suspended for 6 min by adhesive tape about 1 cm from the tip of their tails, and their immobility time was monitored and captured by a video camera. The mice were deemed immobile only if they hung passively or remained completely still.

### 4.10 Hematoxylin and Eosin (H&E) Staining

Mice were all euthanized by inhalation of isoflurane after behavior tests and their brains were collected. Hematoxylin and eosin (H&E) staining was used to analyze the brain morphology. A gradient of alcohol with varying concentrations was used to dewax and dehydrate the paraffin sections of hippocampus tissues. The H&E staining was carried out using an H&E staining kit (GP1031, Servicebio, Wuhan, China) according to the manufacturer's instructions. The sections were submerged in ethanol and xylene, mounted with resin, and observed and photographed under a light microscope (Olympus).

### 4.11 Nissl Staining

Sections of the hippocampus tissues were examined for neuronal damage using Nissl staining. After 30 min of Nissl stain incubation (GP2087, Servicebio, Wuhan, China), the sections were cleaned with 95% ethanol. The Olympus light microscope was used to count and image the labeled cells. On each slide of the hippocampus tissue, cells were counted in three randomly chosen non‐overlapping fields.

### 4.12 SA‐β‐Gal Staining

A commercial β‐galactosidase staining detection kit (G1580, Solarbio, Beijing, China) was used to perform SA‐β‐gal staining. The provided fixative solution was used to fix the sections of the hippocampus and cortical tissues for 15 min at room temperature. Following fixation, the sections were submerged in a brand‐new β‐gal staining solution in buffer and left to incubate for the entire night at 37°C. The Olympus light microscope was used to take microscopic pictures of the stained sections.

### 4.13 Cell Culture

Human microglia cell line HMC3 was purchased from Pricella (CL‐0620, Wuhan, China) and cultured in MEM containing 10% fetal calf serum and 1% penicillin/streptomycin. Cells were seeded in 6‐well plates 1 day prior to experimental treatments. HMC3 cells were divided into 5 groups: the Old EV group treated with 200 μg protein equivalent EVs extracted from plasma of individuals older than 80 years for 24 h, the Old EV + DMSO group treated with old EVs and the same volume of DMSO as the prepared drugs, the Old EV + 3TC group treated with both old EVs and 40 μmol/L 3TC, the Old EV + H151 group treated with old EVs and 1 μmol/L H151, and the control group cultured with an equal amount of PBS as the old EVs.

### 4.14 Immunofluorescence Staining

Cells or frozen mouse brain slices were first cleaned with PBS and treated with 4% paraformaldehyde prior to immunofluorescence examination. After being rendered transparent with PBS containing 0.1% Triton X‐100, the samples were blocked for 1 h at room temperature using a 5% goat serum/PBS solution. Slides were coated with primary antibodies diluted in PBS and incubated overnight at 4°C. Then, the slides were exposed to DAPI for 7 min at room temperature and then secondary antibodies (Alexa Fluor555, Abcam, ab150078, and Alexa Fluor488, Abcam, ab150113) for 50 min. Lastly, a fluorescent inverted microscope (Leica Microsystems, Biberach, Germany) was used to monitor and image the sealed samples. The antibodies used in this study included anti‐IBA1 (OB‐MMS039, Oasis, Hangzhou, China), anti‐LINE1 ORF1P (ab230966, Abcam, USA), anti‐cGAS (26416‐1‐AP, Proteintech, Wuhan, China), anti‐STING (19851‐1‐AP, Proteintech, Wuhan, China), anti‐P21 (GT1032, Invitrogen, USA), and anti‐beta actin (20536‐1‐AP, Proteintech, Wuhan, China).

### 4.15 RNA Extraction and Real‐Time PCR

Trizol (AG21101, Accurate Biotechnology, China) was used to extract the total RNA of brain tissues of each mouse and EVs derived from various organ tissues and cells, which was then reverse‐transcribed into cDNA. The real‐time PCR experiment was performed using SYBR Green Pro Taq HS premixed qPCR Kit (AG11701, Accurate Biotechnology, China) with the primers for each gene tested. GAPDH was used as an internal reference (Tables S2 and S3). For the clinical study, the RNA was extracted from plasma EVs using Total Exosome RNA and Protein Isolation Kit (ThermoFisher, cat4478545, Rockford, USA) and reverse‐transcribed into cDNA using Taqman Reverse Transcription Kit (Applied Biosystems, Carlsbad, USA). The real‐time PCR experiment was performed using specific primers (Table S1), TaqMan probe, TaqMan Fast Advanced Master Mix, and sterilized water, and *hSATA* was used as the endogenous gene. At least three independent technical replicates were performed for each sample to ensure reliability and reproducibility, and the 2^−ΔΔ*Ct*^ method was used to calculate the gene expression levels.

### 4.16 Protein Extraction and Western Blot Analysis

Cells or tissues were lysed with RIPA Lysis Buffer with the addition of protease phosphatase inhibitor mixture (P0013B, Beyotime Biotechnology, China). Total protein was collected, measured, and standardized utilizing the BCA Protein Analysis Kit (PC0020, Solarbio, Beijing, China). Electrophoresis was performed on SDS‐PAGE gel, which was transferred to the PVDF membrane (ISEQ00010, Merck Millipore, China). The membranes were then blocked with 5% skimmed milk or 5% BSA diluted in TBST for 1 h at room temperature. After washing in TBST, the membranes were incubated with primary antibodies overnight at 4°C. Then, the membranes were washed in TBST and incubated with secondary antibodies for 1 h at room temperature. Finally, the protein bands were visualized using Tanon's imaging equipment (Shanghai, China) and the Immobilon Western HRP substrate Luminol reagent (Millipore, USA). ImageJ software (NIH, USA) was used to calculate the optical density of immunoblots. The primary antibodies used in this experiment included those for immunofluorescence, as well as anti‐ALIX (ab186429, Abcam, USA), anti‐GM130 (ab52649, Abcam, USA), anti‐CD63 (A5271, ABclonal, China), anti‐Calnexin (66903‐1‐lg, Proteintech, Wuhan, China), anti‐NeuN (66836‐1‐lg, Proteintech, Wuhan, China), anti‐TBK1 (28397‐1‐AP, Proteintech, Wuhan, China), anti‐pTBK1 (5483T, CST, USA), anti‐IRF3 (A2172, ABclonal, USA), anti‐pIRF3 (AP1412, ABclonal, USA), anti‐pSTING (50907T, CST, USA), anti‐TNFα (60291‐1‐lg, Proteintech, Wuhan, China), and anti‐GFAP (Abcam, ab33922, USA).

### 4.17 Statistical Analysis

The statistical packages R (The R Foundation; http://www.r.project.org; version 3.4.3) and Empower (R) (www.empowerstates.com, X&Y solutions Inc., Boston, Massachusetts, USA) were used for the clinical study analysis. Continuous variables were reported as median, percent 25–percent 75, or mean ± standard deviation (SD). Group differences were examined using the Kruskal–Wallis *H* test and one‐way ANOVA. The correlation between the levels of EV LINE‐1 RNA and plasma brain aging biomarkers was examined using Spearman correlation and multivariable linear regression analysis. For the animal and cellular experiments, data were analyzed using GraphPad and Prism version 9.0 (RRID: SCR_002798). Comparisons between groups were made using unpaired *t*‐tests and one‐way or two‐way ANOVA. The significant difference was determined based on *p* < 0.05.

## Author Contributions

S.Y.: conceptualization, data curation, formal analysis, investigation, methodology, software, writing – original draft. Q.C.: project administration. Q.Y.: project administration. S.W.: data curation. Z.C.: data curation. H.C.: data curation. Y.J.: resources, supervision. Y.W.: resources, supervision. M.L.: conceptualization, methodology, software, writing – original draft, writing – review and editing. Z.L.: writing – review and editing, supervision, funding acquisition.

## Funding

This work was supported by the National Natural Science Foundation of China (82272414), Taishan Scholars Program of Shandong Province (tsqnz20240852), and Jinan Clinical Medical Science and Technology Innovation Plan (202134029).

## Ethics Statement

This study was approved by the Ethics Committee for Biomedical Research Involving Human Beings of Provincial Hospital affiliated to Shandong First Medical University (NSFC: No. 2022‐511). Animal study was approved by the Laboratory Animal Management and Ethics Review Committee of Shandong Provincial Hospital (NSFC: No. 2022‐511).

## Consent

All participants provided written informed consent.

## Conflicts of Interest

The authors declare no conflicts of interest.

## Supporting information


**Data S1:** acel70350‐sup‐0001‐Supinfo.docx.

## Data Availability

The data that support the findings of this study are available on request from the corresponding author. The data are not publicly available due to privacy or ethical restrictions.
