# Supplementary material for: Systemic LINE‐1 RNA in Plasma Extracellular Vesicles Drives Neuroinflammation and Cognitive Dysfunction via cGAS‐STING Pathway in Aging
Source: Aging Cell. 2026 Jan 2;25(1):e70350. doi: 10.1111/acel.70350 (PMC12757926; doi:10.1111/acel.70350)
Supplement: Supplementary file 1 — Data S1: acel70350‐sup‐0001‐Supinfo.docx. [file ACEL-25-e70350-s001.docx]

**
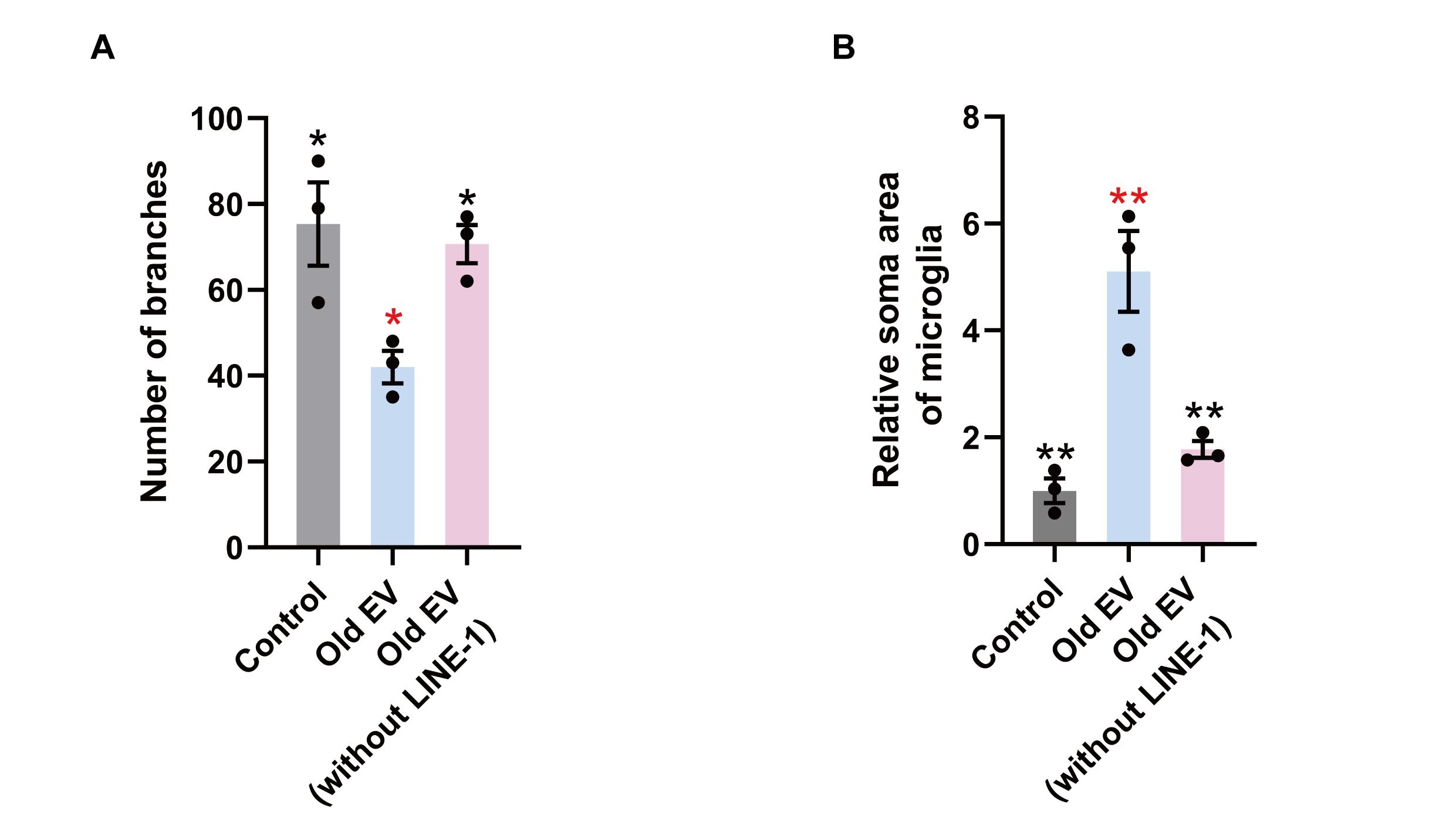
**

**Figure S1.** Morphological analysis of microglia. (A) Morphological analysis of the number of branches of microglia in Figure 4. (B) Morphological analysis of the soma area of microglia in Figure 4.


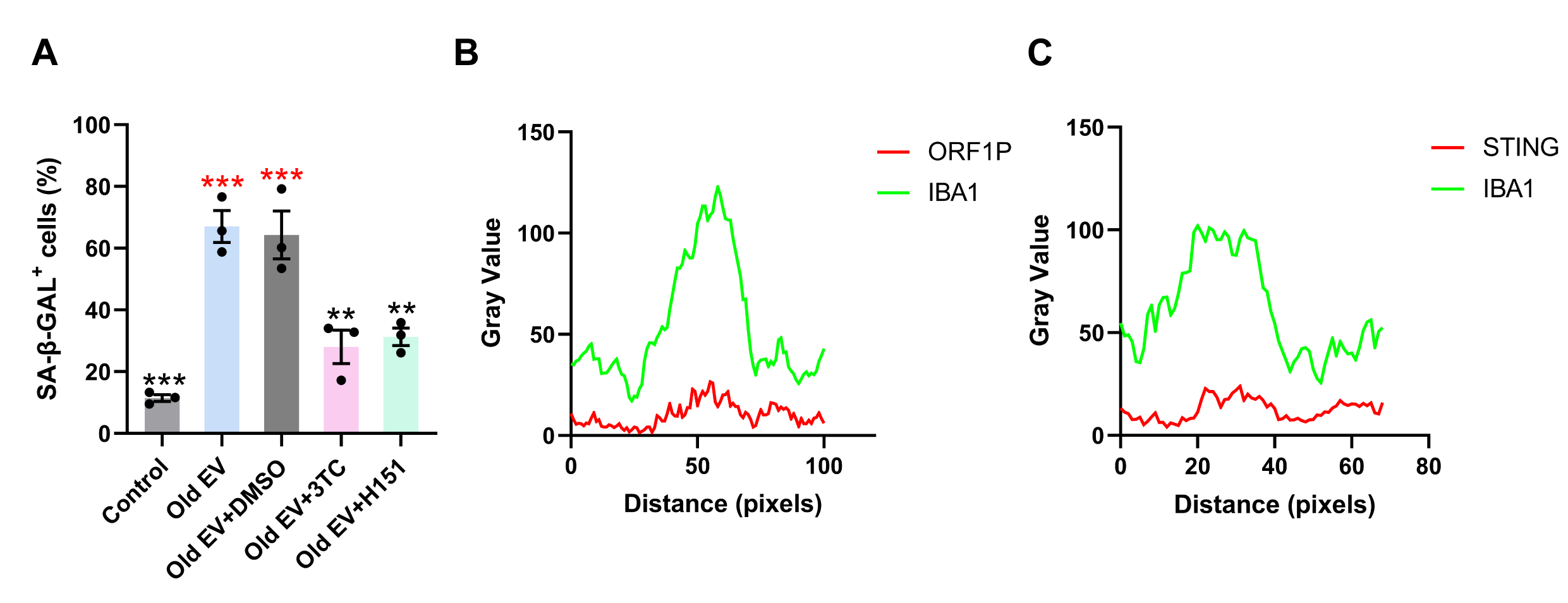


**Figure S2. (A)** Percentage of positive SA-β-gal cells in each visual field in Figure 7E. (B) Colocation analysis of ORF1P (red) and IBA1(green) in Figure 7A. (C) Colocation analysis of P21 (red) and IBA1(green) in Figure 7D.


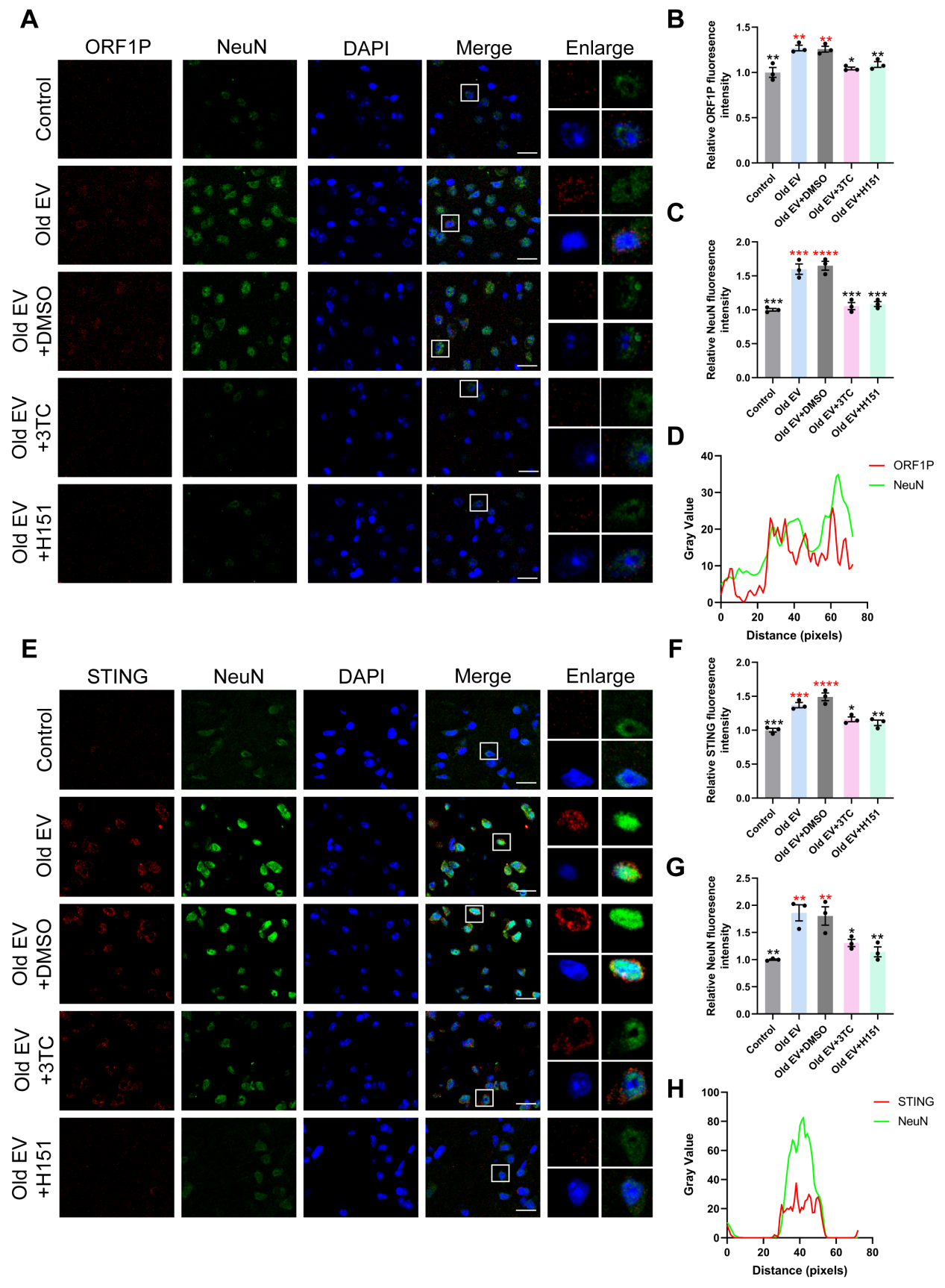


**Figure S3.** Inhibition of LINE-1 reverse transcription or the cGAS/STING pathway ameliorates the old EV-induced neuron activation. (A) Representative images of immunofluorescence detection of LINE-1 ORF1P and NeuN in the mouse brain (scale bar =20 μm). (B, C) Quantification of immunofluorescence analysis presented in (A). (D) Colocation analysis of ORF1P (red) and NeuN (green) in (A). (E) Representative images of immunofluorescence detection of STING and NeuN in the mouse brain (scale bar =20 μm). (F, G) Quantification of immunofluorescence analysis presented in (B). (H) Colocation analysis of STING (red) and NeuN (green) in (A). Black asterisks represent statistical differences compared to the Old EV group. Red asterisks represent statistical differences compared to the control group. One-way ANOVA is used to analyze the differences between groups for normally distributed data, and non-normally distributed data are analyzed by non-parametric tests. Significant difference from control is determined based on *p* < 0.05 (*), *p* < 0.01 (**), *p* < 0.001 (***), and *p* < 0.0001 (****), respectively.


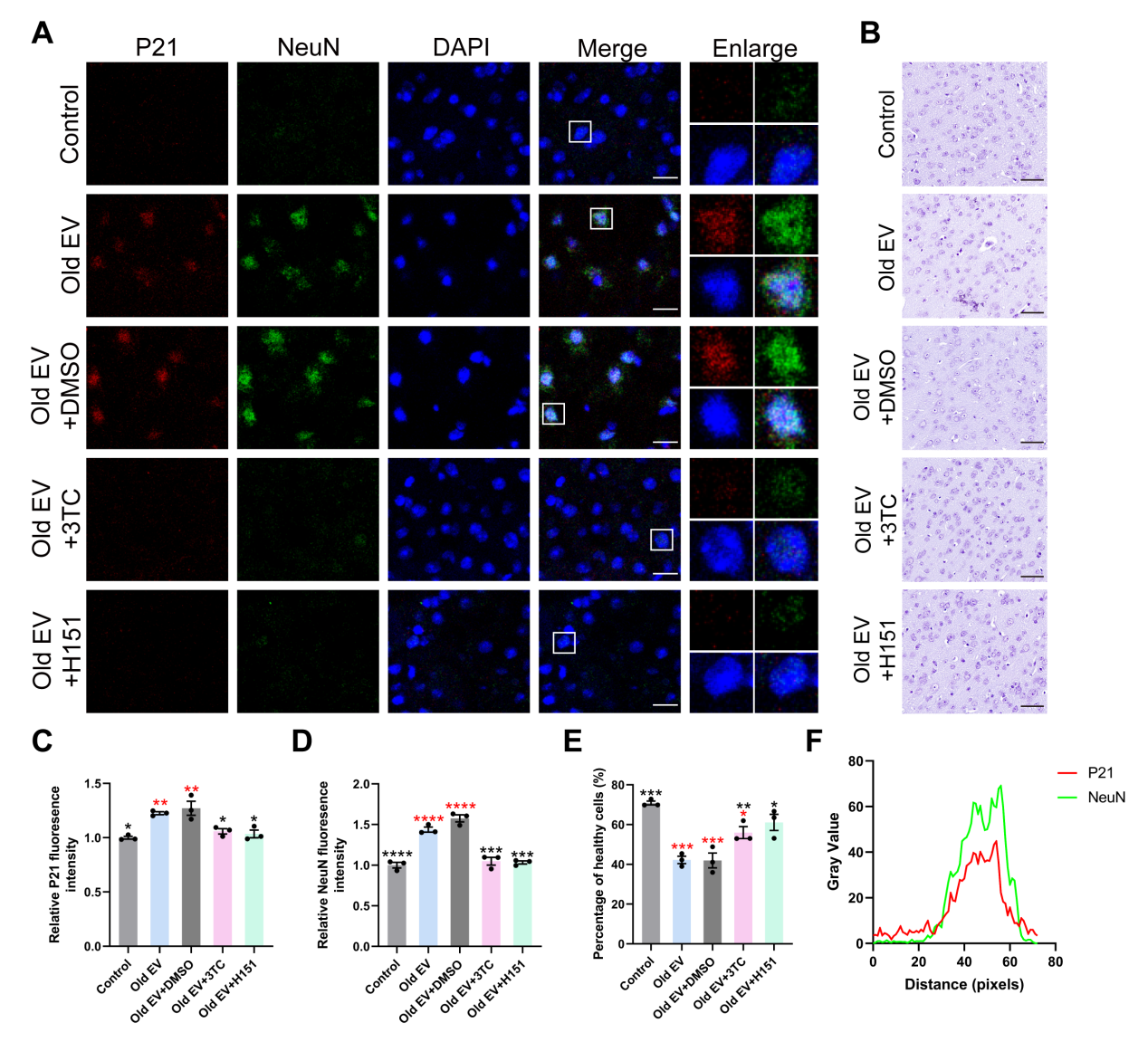


**Figure S4.** Inhibition of LINE-1 reverse transcription or the cGAS/STING pathway ameliorates the old EV-induced neuron senescence. (A) Representative images of immunofluorescence detection of P21 and NeuN in the mouse brain (scale bar =20 μm). (B) Identification of neuronal survival by Nissl staining in the hippocampus (scale bar = 20 μm). (C, D) Quantification of immunofluorescence analysis presented in (A). (E) Percentage of healthy neuronal cells in each visual field with densely stained Nissl bodies (n = 3). (F) Colocation analysis of P21 (red) and NeuN (green) in (A). Black asterisks represent statistical differences compared to the Old EV group. Red asterisks represent statistical differences compared to the control group. One-way ANOVA is used to analyze the differences between groups for normally distributed data, and non-normally distributed data are analyzed by non-parametric tests. Significant difference from control is determined based on *p* < 0.05 (*), *p* < 0.01 (**), *p* < 0.001 (***), and *p* < 0.0001 (****), respectively.

**Table S1.** Primers used for qRT-PCR analysis of EV LINE-1 mRNA in cohort studies. “-F” and “-R” indicate forward and reverse primers, respectively.

| **Primer** | **Species** | **Method** | **Sequence (5'🡪3')** |
| --- | --- | --- | --- |
| h5’UTR | Human | Taqman | PCR primer-F: ACAGCTTTGAAGAGAGCAGTGGTT |
|  |  |  | PCR primer-R: AGTCTGCCCGTTCTCAGATCT |
|  |  |  | Taqman probe: TCCCAGCACGCAGC |
| hORF1 | Human | Taqman | PCR primer-F: GAATGATTTTGACGAGCTGAGAGAA |
|  |  |  | PCR primer-R: GTCCTCCCGTAGCTCAGAGTAATT |
|  |  |  | Taqman probe: AAGGCTTCAGACGATC |
| hORF2 | Human | Taqman | PCR primer-F: CAAACACCGCATATTCTCACTCA |
|  |  |  | PCR primer-R: CTTCCTGTGTCCATGTGATCTCA |
|  |  |  | Taqman probe: AGGTGGGAATTGAAC |
| hSATA | Human | Taqman | PCR primer-F: GGTCAATGGCAGAAAAGGAAAT |
|  |  |  | PCR primer-R: CGCAGTTTGTGGGAATGATTC |
|  |  |  | Taqman probe: TCTTCGTTTCAAAACTAG |

**Table S2.** Primers used for qRT-PCR analysis of EV LINE-1 mRNA in animal studies. “-F” and “-R” indicate forward and reverse primers, respectively.

| **Primer** | **Species** | **Method** | **Sequence (5'🡪3')** |
| --- | --- | --- | --- |
| LINE-1 | Mouse | SYBR Green | PCR primer-F: ACAGCCTCCGTCAGTTTACCA |
|  |  |  | PCR primer-R: ATGCTTCGTGTCTCCTAGCGT |
| GAPDH | Mouse | SYBR Green | PCR primer-F: CAATGAATACGGCTACAGCAAC |
|  |  |  | PCR primer-R: AGGGAGATGCTCAGTGTTGG |

**Table S3.** Primers used for qRT-PCR analysis of EV LINE-1 mRNA in in vitro studies. “-F” and “-R” indicate forward and reverse primers, respectively.

| **Primer** | **Species** | **Method** | **Sequence (5'🡪3')** |
| --- | --- | --- | --- |
| GAPDH | Human | SYBR Green | PCR primer-F: GGAGCGAGATCCCTCCAAAAT |
|  |  |  | PCR primer-R: GGCTGTTGTCATACTTCTCATGG |
| LINE-1 | Human | SYBR Green | PCR primer-F: TAAGGGCAGCCAGAGAGAAA |
|  |  |  | PCR primer-R: GCCTGGTGGTGACAAAATCT |
| cGAS | Human | SYBR Green | PCR primer-F: TAACCCTGGCTTTGGAATC |
|  |  |  | PCR primer-R: TGGGTACAAGGTAAAATGGCTTT |
| TBK1 | Human | SYBR Green | PCR primer-F: TGGGTGGAATGAATCATCTACGA |
|  |  |  | PCR primer-R: GCTGCACCAAAATCTGTGAGT |
| IRF3 | Human | SYBR Green | PCR primer-F: AGAGGCTCGTGATGGTCAAG |
|  |  |  | PCR primer-R: AGGTCCACAGTATTCTCCAGG |
| STING | Human | SYBR Green | PCR primer-F: CCAGAGCACACTCTCCGGTA |
|  |  |  | PCR primer-R: CGCATTTGGGAGGGAGTAGTA |
